# Light-Assisted Formation of Nucleosides and Nucleotides from Formamide in the Presence of Cerium Phosphate

**DOI:** 10.3390/life14070846

**Published:** 2024-07-05

**Authors:** Shoval Gilboa, Larisa Panz, Nitai Arbell, Yaron Paz

**Affiliations:** 1 Department of Chemical Engineering, Technion-Israel Institute of Technology, Haifa 320003, Israel; shovalgilboa@campus.technion.ac.il (S.G.); nitarbell@campus.technion.ac.il (N.A.); 2 The Schulich Department of Chemistry, Technion-Israel Institute of Technology, Haifa 320003, Israel; panz1@technion.ac.il

**Keywords:** origin of life, cerium phosphate (CePO_4_), nucleotides, photocatalysis one-pot reaction

## Abstract

The abiotic formation of nucleotides from small, simple molecules is of large interest in the context of elucidating the origin of life scenario. In what follows, it is shown that nucleosides and nucleotides can be formed from formamide in a one-pot reaction utilizing the mineral cerium phosphate (CePO_4_) as a photocatalyst, a catalyst and a reactant that supplies the necessary phosphate groups. While the most abundant RNA/DNA building blocks were thymidine and thymidine monophosphate, considerable yields of other building blocks such as cytidine, cytidine monophosphate, and adenosine cyclic monophosphate were found. Comparing the yield of nucleosides and nucleotides under light conditions to that in the dark suggests that in the presence of cerium phosphate, light promotes the formation of nucleobases, whereas the formation of nucleotides from nucleosides take place even in the absence of light. The scenario described herein is considerably simpler than other scenarios involving several steps and several reactants. Therefore, by virtue of the principle of Occam’s razor, it should be of large interest for the community.

## 1. Introduction

The RNA world hypothesis postulates that the first step towards the appearance of living entities involves the formation of RNA, since this molecule may not only self-replicate [1] but also may act as a catalyst [2,3]. The hypothesis leans on the concept that under the conditions of the primitive atmosphere on primordial Earth, prebiotic chemistry played a role by increasing the complexity of simple organic molecules to eventually form biological compounds such as RNA chains. Such a process is likely to comprise the following sequence: formation of sub-building blocks (most likely, but not necessarily, nucleobases and ribose) that produce, together with phosphate, building blocks (nucleotides). This is followed by spontaneous polymerization, yielding oligomers of short RNA that may function as non-enzymatic catalysts [2].

The formation of the ribose part in nucleotides is often attributed to the well-known formose reaction starting from formaldehyde and continuing via the formation of glycolaldehyde and glyceraldehyde [4]. However, in that case, more reactants are needed for forming the nucleobases in a separate step. Moreover, the environmental conditions required for the formose reaction to happen are quite different from those vital for the formation of nucleobases. For this reason, the finding that hydrogen cyanide may form sugars in the presence of a carbon source and ultraviolet light provides a plausible alternative route [5].

Formamide, a simple molecule containing the four essential elements (nitrogen, oxygen, hydrogen, and carbon), has been considered as a good candidate for forming life-building blocks [6,7]. Indeed, several studies have shown the formation of amino acids [8], nucleobases [9,10,11], sugars [12,13], nucleosides [14,15] and amino acid derivatives [16] from formamide. Formamide is known to be prevalent in abiotic environments, such as on comets and in outer space, increasing the likelihood of its involvement in the creation of life [17,18]. Parts of the measurements with meteorites were performed in the presence of UV light, which presumably assisted the formation of relevant molecules; nevertheless, complete nucleotides were not reported [19].

A few previous works have shown the formation of nucleotides from existing nucleosides, through the addition of a phosphate group [7,20,21]. Nevertheless, we are not aware of any work demonstrating the formation of a complete nucleotide from primitive precursors in an abiotic one-pot reaction under constant conditions. So, while a certain level of understanding has been obtained about how the different steps in the process may have taken place, science has not yet managed to unfold the whole process [22,23,24].

Since the pioneering direct synthesis of cytidine-monophosphate from glycolaldehyde [25], few examples of the formation of complete nucleotides from primitive precursors have been reported. This final step, grafting a phosphate group onto a nucleoside, requires an available source of sufficiently labile phosphate groups [26]. Accordingly, this step may be reliant on the presence of phosphate minerals, which may serve as a reservoir of these functional groups [27]. An appropriate candidate as a phosphate-donating mineral for prebiotic synthesis is cerium phosphate (CePO_4_). Cerium is the most abundant element among the rare-earth elements [28], making it more abundant than copper and five times more abundant that lead. Many of its compounds are active as catalysts and photocatalysts. Igneous phosphate rocks, which seem to be a key player in the formation of biomolecules, typically contain 1000–32,000 ppm of cerium (there is even a report on apatite rocks containing as much as 11% cerium) [29]. Cerium phosphate appears in the Earth’s crust as the mineral monazite, which, under some conditions, adopts the rhabdophane (CePO_4_·nH_2_O) structure [30]. This common mineral [31] not only contains a phosphate group, which presumably could be donated during the reaction to form nucleotides, but also acts as a low-activity photocatalyst that promotes the formation of electron–hole pairs under UV irradiation, which can be further involved in redox reactions [32,33,34]. For this reason, a scenario in which CePO_4_ plays an essential role in a one-pot reaction leading to formation of nucleotides from the simple molecules seems plausible. Its low photocatalytic activity [33] may potentially even be of benefit, allowing for more delicate reactions to occur without the rapid formation of reactive oxygen species such as hydroxyl radicals, ozone, and atomic oxygen. These species, if formed, could cause the complete oxidation of formed products, since oxidation reactions, being competing downhill reactions, are known to be a major limitation in artificial photosynthesis [35]. Nevertheless, despite the potential advantages of utilizing cerium phosphate as a photocatalyst for synthesizing biomolecules from simple compounds, we are not aware of any work demonstrating its operation in this context.

Another important obstacle in the abiotic formation of biological building blocks, which still puzzles scientists around the world, is the water paradox. On one hand, water is crucial for life; in the absence of water, life is impossible (at least, the sort of life that exists on planet Earth). On the other hand, water is deleterious for the formation of biomolecules [36]. For example, polymers of amino acids (proteins) and polymers of nucleic acids (DNA/RNA) are vulnerable at their joints [37]. This is well reflected in the words of the biochemist Robert Shapiro in his book *Origins*, (cited by Michael, M. 2020 [37]): “…In carbon chemistry, water is an enemy to be excluded as rigorously as possible”. In the context of the RNA world hypothesis, this paradox can be stated as: “RNA requires water to function, but RNA cannot emerge in water, and does not persist in water without repair” [37]. One way to resolve this paradox is by considering a multi-functional catalyst that functions as a reaction promoter, photocatalyst, phosphate donor, and an adsorption site for life-building sub-blocks formed in the process, thus increasing their stability and promoting high concentrations of both nucleobases and sugars in close proximity. This facilitates the formation of more-complex compounds, such as nucleotides and eventually strands of RNA.

In what follows, the formation of RNA/DNA building blocks such as nucleobases, nucleosides, and nucleotides by heterogeneous photoreactions is demonstrated, thus supporting the notion that these (or similar) processes were at the core of the formation of the first biological molecules. To the best of our knowledge, this is the first-ever manuscript detailing the successful formation of complete nucleotides, including a phosphate backbone, in a one-pot reaction, from primitive organic molecules, and without the presence of biological catalysts. The results shown herein may not only help our understanding of how life on Earth could have started but may also be of use in learning how to better implement photocatalytic reactions, typically applied for the aggressive degradation of organic compounds, towards the highly delicate synthesis of fine chemicals.

## 2. Materials and Methods

**Chemicals**—CePO_4_ (CAS:13454-71-2) was purchased from Alfa-Aesar (Ward Hill, MA, USA) and was characterized by XRD (Figure S1, Table S1, revealing rhabdophane structure having a crystallite size of 14.8 nm by the Scherrer equation), UV-vis diffuse reflectance (Figure S2, Table S3) and BET adsorption isotherms (Table S2, 46.4 m^2^/g). Formamide (CAS:75-12-7) was purchased from Merck (Rahway, NJ, USA). All standards used for LC-MS (adenine, thymine, cytosine, uracil, adenosine, uridine, thymidine, guanosine, cytidine, AMP, CMP, TMP, UMP, GMP) were purchased from Sigma-Aldrich (Burlington, MA, USA). The cyclic nucleotide cAMP was purchased from TCI company (Germantown, WI, USA). HPLC-grade water was purchased from Macron LTD (Bologna, Italy).

**Reactions**—Carousel 12+ Reaction Station, Radleys Ltd. (London, UK) was used for the reaction setup. Prior to any reaction, all reaction vessels were cleaned using a piranha solution (Caution!). A 10 mL volume of formamide and 0.264 g of CePO_4_ were introduced to each reaction vessel. The tubes were then heated to 170 °C under stirring and remained at that temperature for 48 h. Nitrogen gas flowed into the reaction vessel continuously at a volumetric flow rate of 0.2 L/min. Half of the samples were irradiated by a UV 365 nm LED (18.6 mW/cm^2^, irradiated area: 1.45 cm^2^), while the other half of the samples were kept in the dark. Following a reaction time of 48 h, the liquid was separated from the catalyst by centrifugation at 14,000 rpm for 10 min. Then, 0.1 mL of the liquid phase was introduced into a rotary evaporator (90 °C, 2 h, under vacuum). Once the solvent had been fully evaporated, a brown crude product was obtained. This crude product was dissolved in 0.5 mL of HPLC-grade water and measured by high-resolution LC-MS.

**Identification of Products**—Identification and quantification of formed DNA/RNA building blocks were performed by LC-MS Maxis impact Bruker (Billerica, MA, USA) with a positive electron spray ionization (ESI) MS method, in two configurations: High-resolution direct MS (HR-MS) and LC-MS. For the LC-MS measurements, two mobile phases were used A: acetonitrile, B: 0.1%*w*/*w* formic acid in HPLC-grade water. The separation sequence started with 100% of B for 5 min at a flow rate of 0.3 mL/min (for all samples), then 1 min with a mixture comprising 99% B and 1% A, 1 min with 95% B and 5% A, 2.5 min with 100% A and at last, 5.5 min with 100% B, based on a previously published procedure [38]. A C18 Luna 5µ column (Phenomenex, Torrance, CA, USA) was used. In each measurement, a volume of 5 µL was injected into the column. The ESI detector conditions were as follows: capillary 4500 V, nebulizer 3.0 bar, source 180 °C and dry gas flow of 8 L/min. Quantification was made by comparing the integrated signals of the samples at a specific *m*/*z* and retention time to those of calibration curves of commercial standards. Such identification methods cannot negate the possibility of having other isomers showing the same retention time. To prevent misunderstanding, an asterisk was added in the figures describing the products. For nucleotides, we used standards in which the phosphate groups were attached at the 5′-position, whereas for cyclic adenosine monophosphate, a standard in which the phosphate group was attached at 3′- and 5′-positions and not at 2′,3′ was used. The nucleobase guanine was not measured due to its low solubility in water.

The high-resolution direct MS measurements served for preliminary identification of the building blocks, as well as for analyzing control experiments used for verifying the lack of contaminants in the formamide and the CePO_4_. Here, a Xevo G2 QTOF (Waters Ltd., Milford, MA, USA), with a positive ESI detector was used. The mobile phase comprised 30% water and 70% acetonitrile at a flow rate of 0.5 mL/min and a sample volume of 40 µL. For the CePO_4_ measurements, the catalyst particles were added into a tube containing HPLC-grade water and heated for 1 h to 90 °C. Then, the liquid was separated from the catalyst by centrifugation at 14,000 rpm for 10 min and measured by HR-MS (see Supplementary Materials).

The identification of the main constituents in the crude mixture of products was performed by GC-MS using an HP 6890N (Agilent, Santa Clara, CA, USA), equipped with an HP5-MS capillary column and a Mass Selective Detector (MSD). Here, a volume of 1 µL of each sample was injected into the GC column that was maintained at 100 °C (for 2 min). The temperature was then raised to 280 °C at 10 °C/min and maintained at this temperature for 20 min. The temperatures of the injector and the detector were set to 280 °C and 300 °C, respectively. Helium (1 mL/min) was used as the carrier gas. Identification was based on a comparison of the retention times and mass spectra with selected commercial compounds (adenine, cytosine, thymine, and uracil—all in formamide). No comparison with guanine was performed, as it does not dissolve well in formamide. Alternatively, a comparison with MS library data (NIST mass spectral library V. 2.0) was performed.

X-ray photoelectron spectroscopy (XPS) measurements of CePO_4_ prior to and following the reaction were performed in an analysis chamber (UHV, 2 × 10^−10^ Torr during analysis) using a Versaprobe III—PHI Instrument (PHI, Phoenix, AZ, USA). The samples were irradiated with a Focused X-Ray AlKα monochromated X-ray source (1486.6 eV) using an X-ray beam (size 100 μm, 25 W, 15 kV). The outcoming photoelectrons were directed to a Spherical Capacitor Analyzer (SCA). The sample charging was compensated by a dual beam charge neutralization based on a combination of a traditional electron flood gun and a low-energy argon ion beam.

**Adsorption Experiments**—First, 3.7 mM solutions of each one of the compounds (adenine, adenosine, AMP, or cAMP) in formamide were prepared under stirring. A 4 mL volume of solution was added into a tube together with 105.2 mg CePO_4_, (2% by weight). The tubes were held in the dark, under stirring and nitrogen flow (0.2 L/m), at a temperature of 300 K. Samples (70 µL) were taken at 0, 10, 30, 60, 90, and 120 min from the beginning of the process. The samples were centrifuged (10,000 rpm, 10 min.) to separate the liquid from the particulate matter. A 25 µL volume of the liquid phase was placed in a clean Eppendorf together with 15 µL of formamide and 1.96 mL of HPLC-grade water. The water was added to stabilize the absorption curve of adenine, whose UV-vis absorption spectrum is known to depend on its charge [39]. The UV-vis absorption of said solution was measured using a Shimadzu UV-2600 spectrophotometer to determine the concentration of the species of interest in the solution. Accordingly, all calibration curves (Figures S32–S35) were prepared with the same water:formamide ratio (1.96 mL water + 0.04 mL formamide).

## 3. Results and Discussion

**Figure 1:**
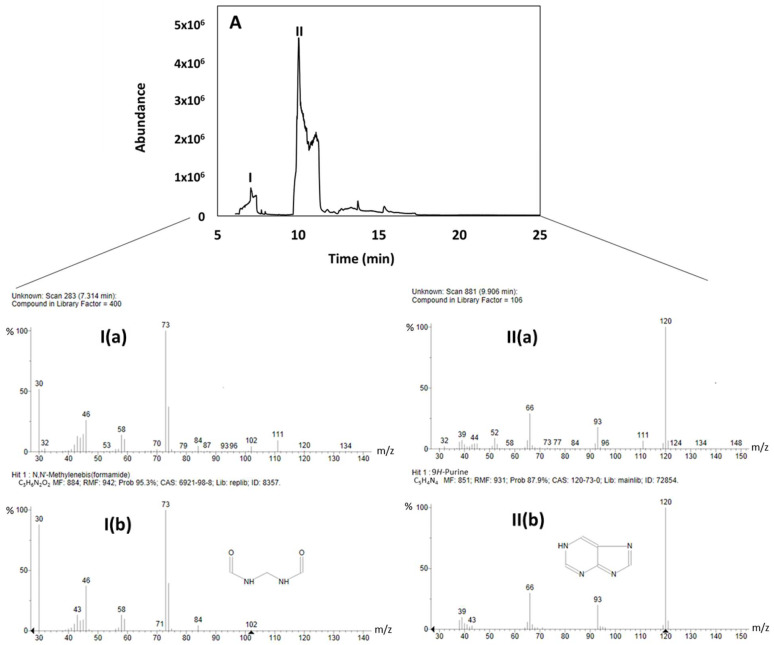
(**A**) GC-MS data of the brown crude product revealing peaks I and II. (**I(a)**) fragmented MS signal of peak I, (**I(b)**) fragmented MS of N,N’-Methylenebis (formamide), (**II(a)**) fragmented MS signal of peak II, (**II(b)**) fragmented MS signal of purine.

A brown viscous mixture of products, termed hereby as “crude” (13 ± 3% of the initial formamide mass), was obtained following 48 h of illumination in the presence of the CePO_4_ particles. The obtained crude was measured by GC-MS (Figure 1A). As shown in the figure, the main constituents of the crude were N,N’-Methylene-bis-formamide and purine.

**Figure 2:**
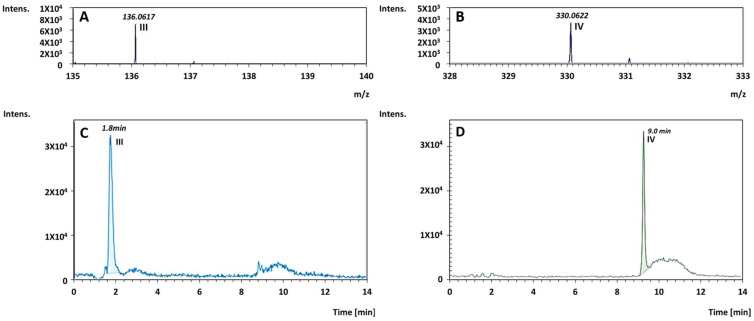
(**A**,**B**) HR direct MS signal, revealing adenine* (**III**) and adenosine cyclic monophosphate (cAMP)* (**IV**), respectively. (**C**,**D**) LC-MS extract-mass signals of the products adenine* and cAMP*, respectively, as found upon performing the reaction under light, in the presence of cerium phosphate. The relevant standards are given in Figure S44B–F in Supplementary Materials. * The asterisk sign represents complete compatibility with nominal commercial standards. The possibility of isomers having same retention times cannot be excluded.

**Figure 3:**
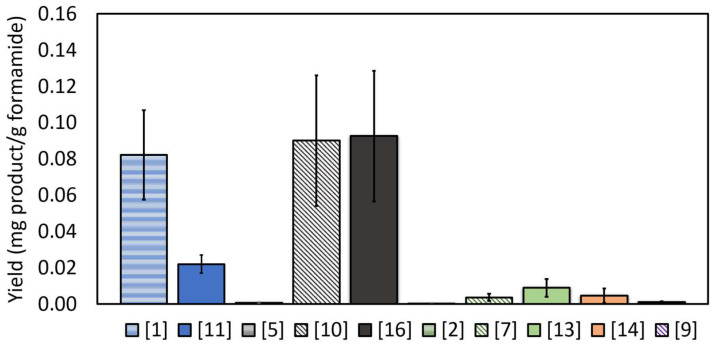
The formation of nucleobases, nucleosides, and nucleotides from formamide in the presence of CePO_4_ and under UV irradiation. Adenine species are shown in blue, thymine in gray, cytosine in green, guanine in orange, and uridine in purple. Nucleobases are presented by horizontal lines, nucleosides by diagonal lines and nucleotides by filled color bars. The numbers in the legend correspond to the molecules listed in Figure S46. ([1] = adenine*, [11] = adenosine cyclic monophosphate*, [5] = thymine*, [10] = thymidine*, [16] = thymidine monophosphate*, [2] = cytosine*, [7] = cytidine*, [13] = cytidine monophosphate*, [14] = guanosine monophosphate*, [9] = uridine*). * The asterisk sign represents complete compatibility with nominal commercial standards. The possibility of isomers having same retention times cannot be excluded.

High-resolution direct MS and LC-MS measurements (Figure 2A–D, Figures S3–S16, S23–S31 and S44), performed on the products, revealed that the crude also contained considerable amounts of other compounds, assigned as nucleobases, nucleosides and even nucleotides. It should be noted that in the case of nucleosides and nucleotides, the regio-chemistry and stereochemistry of the reaction products, as well as the position of the phosphate group on the sugar moiety, were not evaluated and require further investigations. The yield of these species, in terms of mg of product per gram formamide (averaged over 8 repetitions) is given in Figure 3. As shown in the figure, the main RNA/DNA building blocks that were obtained were the nucleoside and the nucleotide of thymine (0.09 mg/g formamide). It is possible, albeit not verified herein, that these compounds were obtained from their uracil homologs by an electrophilic addition of formaldehyde (that was generated from formamide through reduction) at the C-5 position of the uracil ring [11]. This yield is 2 orders of magnitude larger than the yield measured upon using formamide in the presence of various iron-containing meteorites [14]. Apart from these compounds, the nucleobase and the cyclic nucleotide of adenine, as well as the nucleoside and nucleotide of cytosine were found at a yield of 0.0036–0.08 mg/g formamide. In addition, traces of uridine, guanosine monophosphate, cytosine and thymine, at concentrations below accurate quantification limits, were found. It should be noted that these building blocks were obtained at a considerable yield in a one-pot reaction, without altering the conditions during its progression, and without optimizing the reaction time, a parameter that was not investigated in the presented work and is currently under study.

In the past, it was shown that formylated nucleobases and formylated carbohydrates can be formed from formamide/ammonium formate. These can be transformed by known chemistry into nucleosides, nucleotides and partially formylated oligomeric RNA [40]. While the presence of the formylated compounds was not searched by us, due to lack of appropriate standards, we believe that such compounds could indeed be formed. Another issue is the large difference between the high concentrations of thymidine and thymidine monophosphate versus that of uridine, which seems quite odd, considering the resemblance between thymine and uracil. Here, it might be that part of the difference was due to mis-identification of thymidine, being 5-hydroxymethy(deoxy)uridine (the deoxy is needed to maintain the same mass). These two subjects are currently under study.

A set of measurements was performed to negate the possibility of artefacts. The formamide used for the reaction was measured prior to the photocatalytic reaction by MS. None of the above-mentioned compounds were found. In addition, the CePO_4_ catalyst was added to HPLC-grade water and heated to 90 °C for 1 h. Then, the liquid phase was separated from the catalyst by centrifugation and measured by MS. Here, again, no RNA/DNA building blocks were detected (see Supplementary Materials, Figures S17–S22).

**Figure 4:**
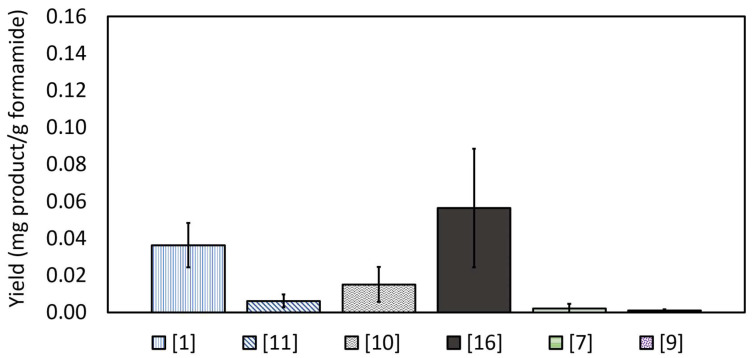
The synthetic yields of life-building blocks from formamide in the presence of CePO_4_ in the dark. Adenine species are shown in blue, thymine in gray, cytosine in green, and uridine in purple. Nucleobases are presented as horizontal lines, nucleosides as diagonal lines and nucleotides as full color bars. The numbers in the legend correspond to the molecules listed in Figure S46. ([1] = adenine*, [11] = adenosine cyclic monophosphate*, [10] = thymidine*, [16] = thymidine monophosphate*, [7] = cytidine*, [9] = uridine*). * The asterisk sign represents complete compatibility with nominal commercial standards. The possibility of isomers having same retention times cannot be excluded.

Another set of experiments served to resolve the contribution of each parameter (catalyst and light) to the formation of the RNA/DNA building blocks. Figure 4 depicts the distribution of RNA/DNA building-block products upon performing the same procedure in the absence of UV light. It was found that the average weight of the obtained crude was not altered significantly with respect to the previous case (14 ± 3 mg per 0.1 mL of liquid), suggesting that the UV light was not the main reason for the formation of N,N’-Methylene-bis-formamide and purine. Nevertheless, when it comes to the formation of RNA/DNA building blocks, significant differences between the two cases were noticed. First, under dark conditions, the overall yield of RNA/DNA building blocks was significantly (20–40%) lower than the yield obtained under light. Second, cytidine monophosphate (CMP), found under light, was not observed in the dark. Third, cytosine, thymine, and guanosine monophosphate, found at sub-quantification concentration under light, could not be observed when the reaction took place in the dark. Hence, it can be concluded that UV light played an important role in increasing the yield and the diversity of the RNA/DNA building blocks formed during the one-pot reaction.

**Figure 5:**
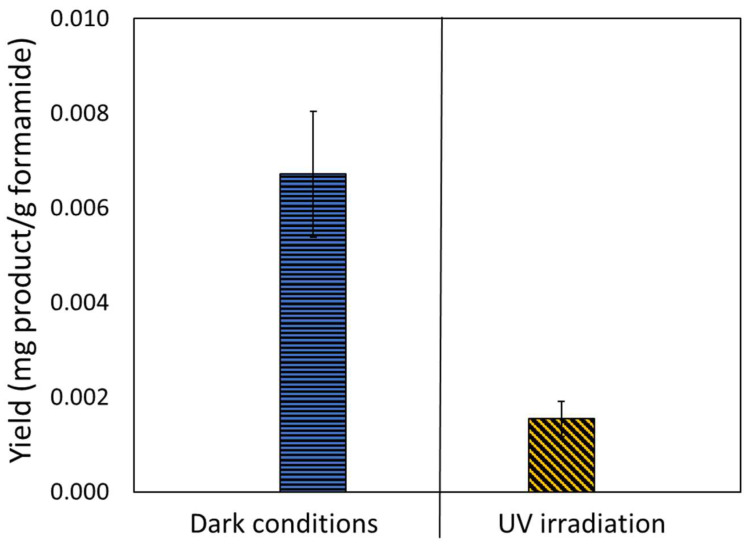
Adenine* formed upon performing the reaction for 48 h in the absence of a catalyst, under dark conditions (left) and upon exposure to UV light (right). No other RNA/DNA building blocks were found. * The asterisk sign represents complete compatibility with nominal commercial standards. The possibility of isomers having same retention times cannot be excluded.

The reaction was run under the same conditions but without the presence of a catalyst. Under both dark and light conditions, the only RNA/DNA building block to be formed was adenine (Figure 5), both at yields considerably lower than the yield in the presence of cerium phosphate. The formation of adenine can be rationalized by a high temperature-driven dehydration of formamide yielding HCN, which is oligomerized subsequently. Evidently, in the absence of cerium phosphate, the yield in the dark was significantly higher than that under light, suggesting that in the absence of a catalyst and under the experimental conditions, light does not play a role in the formation of adenine from formamide, and, in fact, might be deleterious.

**Figure 6:**
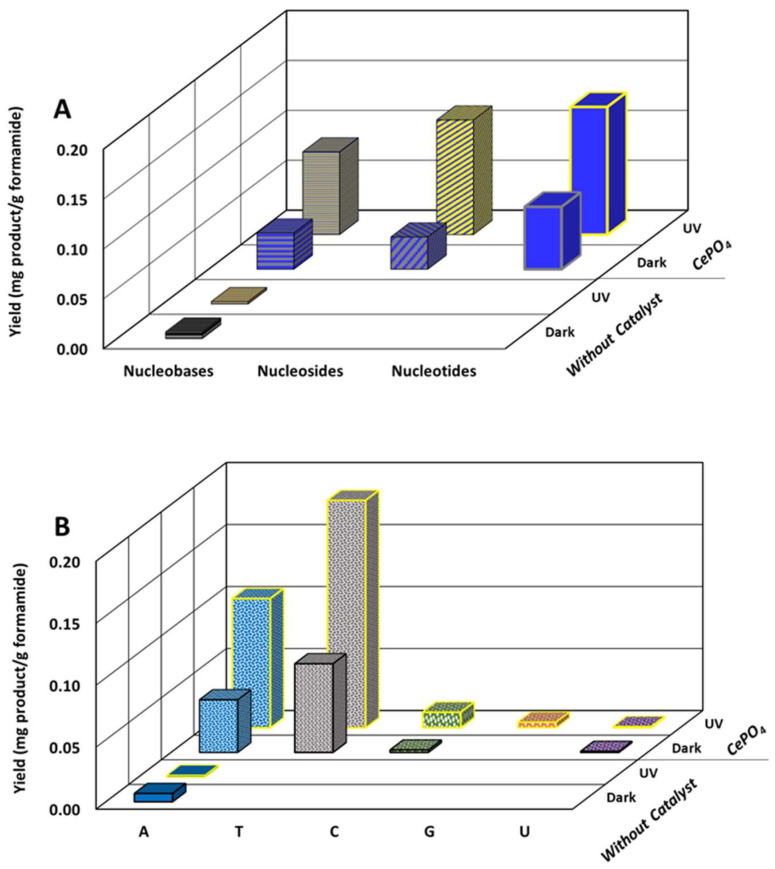
(**A**) The total yield of different forms of the RNA/DNA building blocks, categorized according to nucleobases, nucleosides and nucleotides, as obtained after 48 h with and without CePO_4_ and UV irradiation. (**B**) The total yield of RNA/DNA building blocks (nucleobases + nucleosides + nucleotides) categorized according to the type of nucleobases, as obtained after 48 h with and without CePO_4_ and UV irradiation. A, T, C, G and U represent compounds that contain adenine, thymine, cytosine, guanine and uracil groups, respectively.

An assessment of the role that cerium phosphate plays in the reaction of formamide can be obtained by summing up the yield of all nucleobases and comparing it to the yield of nucleosides and nucleotides (Figure 6A). Such a comparison clearly shows that CePO_4_ acts not only as a reactant, supplying phosphates to form nucleotides, but also as a catalyst, catalyzing the formation of nucleobases and nucleosides. It is further noted that the formation of the more advanced species, nucleosides and nucleotides, did not come at the expense of the formation of nucleobases. On the contrary, the yield of nucleobases in the presence of cerium phosphate was higher than in the absence of CePO_4_, regardless of whether the reaction occurred in the dark or under exposure to light.

Our results revealed the formation of ribose-containing compounds (nucleosides and nucleotides) in the presence of CePO_4_ both in the dark and under light. In contrast, these compounds were not observed in the absence of cerium phosphate. Therefore, it can be deduced that under the experimental conditions (formamide, no oxygen, 170 °C), cerium phosphate acts as a catalyst for the formation of ribose from formamide. It may be noted, however, that previous works have shown that formamide may be converted into formaldehyde [41], which acts as a precursor for the formation of sugars, including ribose, by the Butlerov reaction [4,42], so that the importance of cerium phosphate is in its ability to assist the formation of sugar-containing compounds such as nucleosides and nucleotides rather than promoting the formation of sugars.

Figure 6B presents the total yield of each of the nucleobases formed in the presence of cerium phosphate (under light and in the dark), as well as the overall yield in the absence of cerium phosphate. Here, the total yield of each base was calculated by summing up the contribution of nucleobases, nucleosides, and nucleotides. The figure makes it clear that while the contribution of CePO_4_ to the formation of adenine is relatively mild, the effect of cerium phosphate on the formation of the oxygen-containing nucleobases, in particular the thymine, is dramatic. Thus, our results suggest that CePO_4_, and probably similar phosphate-containing compounds, are essential for the formation of the variety of nucleobases necessary for obtaining nucleotides and eventually RNA and DNA. It is likely that the release of phosphate groups from the cerium phosphate is accompanied by the formation of islands of cerium oxide at the surface. In that case, the documented ability of cerium oxide to catalyze amines to amides [43] may explain the tendency to form the amide-containing nucleobases, in particular thymine and cytosine.

In the presence of cerium phosphate, the relative effect of light is larger on the formation of nucleosides than on the formation of nucleotides, while in the dark, the yield of the nucleotides is considerably larger than that of nucleosides; under light conditions, the yield of formed nucleosides is equal to that of formed nucleotides. This may be explained by a photocatalytic effect on the formation of nucleobases (mostly A and T) that eventually boosts the kinetics of the next step, i.e., the generation of nucleosides.

As reported above, the main compound in the crude mixture of products was purine. The formation of this molecule from formamide requires 5 formamide molecules (in the absence of another carbon source) or 4 molecules (in the absence of another nitrogen source). This means that the formation of purine is coupled with the release of oxygen and hydrogen atoms, which are essential for the formation of sugars, among which are pentoses such as ribose.

In all cases (i.e., with and without light, with and without cerium phosphate) the crude mixture had a brown color (probably due to the formation of polymerized sugars) and in fact was absorbing at 365 nm. As such, the progressing change in the UV-vis spectrum during the reaction could affect the photocatalytic production of RNA/DNA building blocks by blocking the 365 nm light used for exciting the CePO_4_.

**Figure 7:**
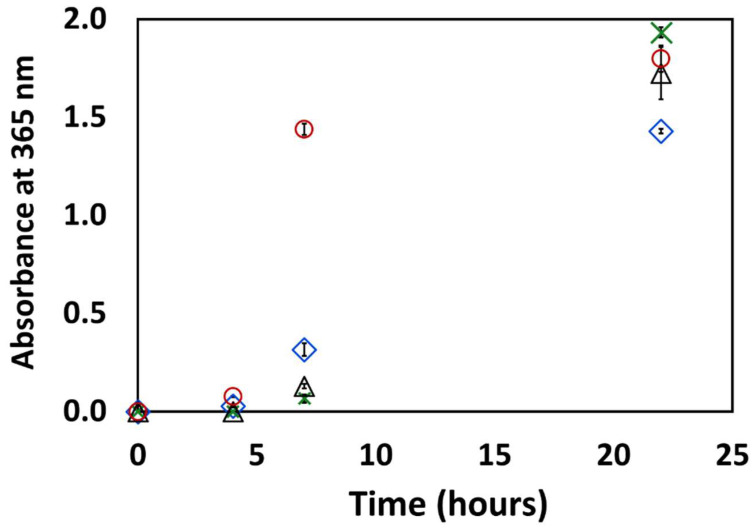
The change in the absorption of the solution at 365 nm during the propagation of the reaction. Red circles: formamide under dark conditions, black triangles: formamide under UV light, green crosses: formamide in the presence of CePO_4_ under dark conditions, blue diamonds: formamide in the presence of CePO_4_ under UV irradiation.

Therefore, the change in the absorption at 365 nm was measured along the evolution of the reaction. The results are given in Figure 7. The figure clearly shows that the formation of the UV-blocking crude is induced by the high temperature of the formamide and not by the presence of cerium phosphate, or by light. In fact, exposure to UV light seems to slow down the formation of the crude mixture. No matter whether CePO_4_ was present or not, and whether UV light was introduced or not, within 24 h, the contents in the reactor practically absorbed almost all impinging photons, so that for the results presented herein, the photocatalytic contribution to the formation of the RNA/DNA building blocks was limited to the first 10–20 h.

**Figure 8:**
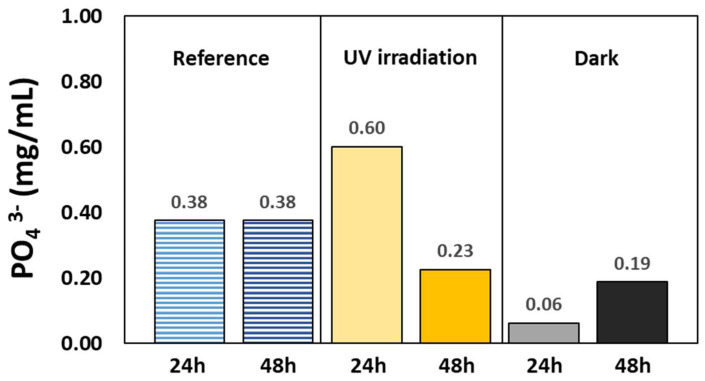
The concentrations of phosphate ions in a mixture of formamide after 24 h and 48 h of reaction at 170 °C, under illumination and in the dark. The reference bars represent measurements performed at room temperature in the dark. In all cases, the initial amount of cerium phosphate was 26.4 mg/mL formamide, equivalent to 10.7 mg phosphate per ml formamide.

The amount of phosphate ions in the liquid was measured by separating the particles, diluting in water, extracting the organics in hexane (seven times), and measuring the amount of phosphate in the aqueous phase by the molybdenum blue method. Results were compared with a reference experiment (formamide + CePO_4_ w/o heating). The results are given in Figure 8. Considerable amounts of phosphates were found, not only upon exposing the system to the conditions prevailing during the reaction (170 °C, under nitrogen, with and w/o light), but also at room temperature (both under dark and illuminated conditions). Apparently, the presence of formamide is sufficient to leach out phosphate groups from cerium phosphate. Upon performing the reaction at elevated temperature, it was found that the concentration of phosphate was significantly higher when the reaction mixture was exposed to UV light than when the reaction took place in the dark. Apparently, light plays a role (directly or indirectly) in the release of phosphate groups. The concentration of phosphate ions after 48 h of reaction under light was lower than after 24 h of reaction (but still higher than the concentration measured when the reaction took place under dark conditions). This may indicate that phosphate groups are first liberated from the particles, and only then, phosphorylation takes place, thus reducing the concentration of phosphate ions in the liquid.

XPS measurements performed by us on the CePO_4_ particles (Figure S45 and Table S6) revealed that following the reaction, an increase in the Ce/P atomic ratio was observed, regardless of whether light was involved or not. This decrease in the ratio may reflect the liberation of phosphate, while maintaining cerium on the surface, for example in the form of CeO_2_ [44]. The XPS results indicated the presence of nitrogen on the surface of the particles following reaction. Higher atomic concentrations were found upon performing the reaction in the dark (9.1 At% and 10.2 At% after 24 h and 48 h of reaction) than upon performing the reaction under UV light (2.3 At% and 6.3 At%, respectively). Correlating these results with the measurements of phosphates in the liquid suggests that nitrogen attached to the surface (CeN ?) plays a role in partial prevention of leaching of phosphate ions from the particles.

Our XPS results did not show any evidence for the presence of Ce(IV), i.e., for the formation of CeO_2_. This finding suggests that formaldehyde, required for the formation of ribose (and other sugars), was not formed by direct reduction of formamide (accompanied by oxidation of Ce(III) to Ce(IV)) but rather by disproportionation of two formamide molecules into one formaldehyde molecule and one carbon dioxide molecule.

It is important to note that the dissolution of cerium phosphate is considerably enhanced in the presence of organic species that may chelate either the cerium ion or the phosphate [45,46]. The high temperatures and high concentration of formamide are likely to enhance the formation of such species, which eventually may assist the release of the phosphate groups. For our particular case, the finding that polysaccharides may increase the rate of dissolution of apatite, a phosphate-containing mineral [44], may suggest a secondary role for the sugars formed during the reaction. It should be noted that there are reports on a possible role of light in the formation of pre-biotic compounds: for example, the formation of guanine, adenine, and hypoxanthine in UV-irradiated formamide solutions [47] and the regioselective synthesis of amino acid-decorated imidazole, purine and pyrimidine derivatives [48]. In fact, there are reported cases in which photocatalysis was used for this purpose. For example, the formation of 6 of the 11 carboxylic acid intermediates of the reductive version of the citric acid cycle was obtained by UV irradiation of formamide in the presence of titanium dioxide [49]. And yet, to the best of our knowledge, this is the first report in which photocatalysis takes part in a complex process that produces not only nucleobases or nucleosides but a variety of nucleotides in a one-pot reaction under constant conditions.

**Figure 9:**
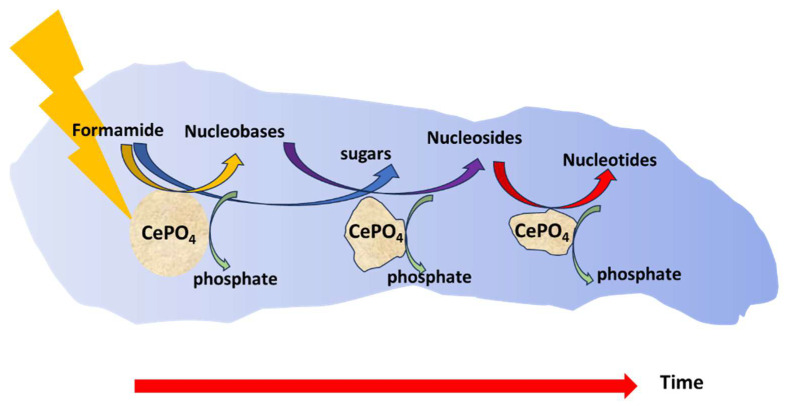
The proposed timeline.

Combining the data depicted in Figure 6 and Figure 7 suggests, but does not prove, a timeline in which formation of nucleobases takes place predominantly by photocatalysis during the first 10–20 h or so. The process continues in the dark by the growth of nucleosides and eventually by the formation of nucleotides while partially consuming the phosphate groups of the photocatalyst. This timeline is portrayed in Figure 9.

One of the main disadvantages of the homogeneous-phase scenario for the origin of life is the inherent difficulty in explaining close proximity between different sub-building blocks (nucleobases, sugars, phosphate) required for the formation of nucleotides and eventually also for their polymerization. A common explanation is the presence of cycles of wet and dry conditions, in which concentrations increase by virtue of evaporation [50,51]. This reasoning is challenged by the discrepancy between the long times associated with such cycles and the limited stability of the sub-building blocks. Moreover, evaporation increases the concentration of all non-volatile substances, including chemical species that are deleterious for the formation of nucleotides, either by way of competition or by way of limiting access.

This drawback is resolved, at least partially, if the formation of all sub-building blocks takes place in close proximity on the surface of particles, and if the involved compounds weakly adsorb on the surface. In that way, high concentrations of reactants are maintained, thus significantly increasing the odds for the formation of nucleotides. By the same token, adsorption of nucleotides (albeit not too strong) paves the way for the necessary polymerization. To verify that cerium phosphate is adequate also from the aspect of adsorption, we have measured the adsorption of adenine, adenosine, adenine-monophosphate (AMP) and 3′-5′-cyclic adenine-monophosphate (cAMP) on cerium phosphate under a formamide environment, mimicking the conditions during the preparation of these compounds (although at lower temperatures, due to technical reasons). The measurement time was limited to two hours in order not to interfere with the formations of other species.

The results, depicted in the Supplementary Materials (Figures S36–S39 and Table S5), reveal that the nucleobase, nucleoside and nucleotide of adenine tend to be adsorbed on the surface of cerium phosphate. As expected, the adsorptivity of these species was found to increase with the size of the species in the order adenine < adenosine < adenosine monophosphate, i.e., with the molecular mass of the species. The only exception was cyclic adenosine monophosphate, whose adsorptivity was found to be very low. The adsorbed amounts, in the order of 10^−7^–10^−8^ moles per m^2^ of catalyst, are quite considerable and correspond, under conservative assumptions, to a coverage of 0.5–48% of the surface area of the catalyst at 300 K. While these values were obtained in the absence of any competition from other species on the adsorptive sites, they are still of importance, as they indicate that the adsorption of sub-building blocks, enroute to the formation of nucleotides (and eventually their polymerization), cannot be ruled out.

## 4. Conclusions

The one-pot formation of nucleobases, nucleosides and even nucleotides from formamide at elevated temperatures and under exposure to UV light was found to be feasible in the presence of cerium phosphate. The cerium phosphate acts largely as a weak photocatalyst during the first hours of the reaction to form nucleobases. At a later stage, it acts mainly as a catalyst for forming nucleosides and eventually contributes phosphate groups for the formation of nucleotides. One of the most intriguing findings is the formation of a large variety of RNA/DNA building blocks, beyond the easy-to-form adenine. It was shown that heterogeneous (photo)catalysis on cerium phosphate may provide adequate conditions for nucleotides’ formation and eventually for polymerization, by virtue of adsorption, leading to the high local concentrations required for RNA/DNA formation.

The results shown herein with cerium phosphate point to a possible role that heterogeneous photocatalysis could have played in the formation of the first biomolecules. In that sense, it is important to outline the basic attributes of this approach, namely a combination of relatively weak photocatalyst, a possibility of donating phosphate groups, an oxygen-free atmosphere and the use of formamide as a reactant.

The demonstration of the formation of nucleotides by a one-pot reaction is of large importance, as this scenario is considerably much simpler than other scenarios involving several steps and/or several reactants. Adopting the principle of Occam’s razor, according to which one should prefer the simplest solution to a problem (alternatively, the solution that requires the smallest possible set of parameters), the approach presented herein should be of large interest to any scientist studying the origin of life, particularly since this scenario is not necessarily unique for cerium phosphate but may be relevant to other phosphate-containing minerals. Work along this line is underway.

## Data Availability

The original contributions presented in the study are included in the article/Supplementary Material, further inquiries can be directed to the corresponding author/s.

## Supplementary Materials

The following supporting information can be downloaded at: https://www.mdpi.com/article/10.3390/life14070846/s1, the supporting information includes characterization of the photocatalytic particles (Figures S1 and S2), Direct-MS crude results (Figures S3–S22), LC-MS calibration curves (Figure S23), Part of the LC-MS crude results (Figures S24–S31), UV-vis calibration curves (Figures S32–S35), Adsorption kinetics (Figures S36–S39), Change in turbidity during the reaction (Figures S40–S43), LCMS standards (Figure S44) and XPS results (Figure S45). Additional information on various RNA/DNA building blocks discussed in this manuscript (Figure S46). Additional information on high-resolution direct-MS measurement and TIC (Figures S47–49).

## Abbreviations

CePO_4_, Cerium phosphate; cAMP, Adenosine cyclic monophosphate; AMP, Adenosine monophosphate; TMP, Thymidine monophosphate; CMP, Cytidine monophosphate; GMP, Guanosine monophosphate; UMP, Uridine monophosphate.
