# Supplementary material for: Light-Assisted Formation of Nucleosides and Nucleotides from Formamide in the Presence of Cerium Phosphate
Source: Life (Basel). 2024 Jul 5;14(7):846. doi: 10.3390/life14070846 (PMC11277737; doi:10.3390/life14070846)
Supplement: Supplementary file 1 [file life-14-00846-s001.zip › life-3037717-supplementary.pdf]

Cerium Phosphate-Assisted Formation of Nucleosides and Nucleotides from Formamide in a One-Pot (Photo)Catalytic Reaction

Shoval Gilboa[1], Larisa Panz,[2] Nitai Arbell[1], Yaron Paz[1,*]

[1] Department of Chemical Engineering, Technion-Israel Institute of Technology, Haifa 320003, Israel

[2] The Schulich Department of Chemistry, Technion-Israel Institute of Technology, Haifa 320003, Israel,

Supporting Information

List of Figures

1. Catalyst Characterization - $CePO_4$

Figure S. 1. XRD pattern of the catalyst $CePO_4$

Figure S.2. (A) The UV-vis diffuse reflectance spectrum of $CePO_4$, (B) Tauc plot assuming indirect transition $[(hvA)^2$ VS. photon energy], (C) Tauc plot assuming direct transition $[(hvA)^{0.5}$ VS. photon energy]

2. Products Identification Using HR-direct MS

2A. HR-direct MS Results of Life-Building Blocks After the One-Pot Reaction of Formamide and $CePO_4$

Figure S.3. HR-direct MS results of the nucleobase guanine found in the reactor vessel following 48 hrs. of reaction at 170°C under light and in the presence of cerium phosphate.

Figure S.4. HR-direct MS results of the nucleobase cytosine found in the reactor vessel following 48 hrs. of reaction at 170°C under light and in the presence of cerium phosphate.

Figure S.5. HR-direct MS results of the nucleobase adenine found in the reactor vessel following 48 hrs. of reaction at 170°C under light and in the presence of cerium phosphate.

Figure S.6. HR-direct MS results of the nucleobase uracil found in the reactor vessel following 48 hrs. of reaction at 170°C under light and in the presence of cerium phosphate.

Figure S.7. HR-direct MS results of the nucleoside cytidine found in the reactor vessel following 48 hrs. of reaction at 170°C under light and in the presence of cerium phosphate.

Figure S.8. HR-direct MS results of the nucleoside adenosine found in the reactor vessel following 48 hrs. of reaction at 170°C under light and in the presence of cerium phosphate.

Figure S.9. HR-direct MS results of the nucleoside uridine found in the reactor vessel following 48 hrs. of reaction at 170°C under light and in the presence of cerium phosphate.

Figure S.10. HR-direct MS results of the nucleoside thymidine found in the reactor vessel following 48 hrs. of reaction at 170°C under light and in the presence of cerium phosphate.

Figure S.3. HR-direct MS results of the nucleoside guanosine found in the reactor vessel following 48 hrs. of reaction at 170°C under light and in the presence of cerium phosphate.

Figure S.4. HR-direct MS results of the cyclic nucleotide cAMP found in the reactor vessel following 48 hrs. of reaction at 170°C under light and in the presence of cerium phosphate.

Figure S.5. HR-direct MS results of the cyclic nucleotide cGMP found in the reactor vessel following 48 hrs. of reaction at 170°C under light and in the presence of cerium phosphate.

Figure S.6. HR-direct MS results of the nucleotide TMP found in the reactor vessel following 48 hrs. of reaction at 170°C under light and in the presence of cerium phosphate.

Figure S.7. HR-direct MS results of the nucleotide GMP found in the reactor vessel following 48 hrs. of reaction at 170°C under light and in the presence of cerium phosphate.

Figure S.8.HR-direct MS results of the nucleotide CMP found in the reactor vessel following 48 hrs. of reaction at 170°C under light and in the presence of cerium phosphate.

2B. HR-direct MS Results of Formamide and Water Following Heating in the presence of $CePO_4$

Figure S.17. HR-direct MS results of formamide as received. Each trace depicts the temporal profile of a specific mass of interest, demonstrating the lack of products in the formamide prior to the reaction.

Figure S.9. HR-direct MS results of formamide (continued). Each trace depicts the temporal profile of a specific mass of interest, demonstrating the lack of products in the formamide prior to the reaction.

Figure S.19. HR-direct MS results of formamide (continued). Each trace depicts the temporal profile of a specific mass of interest, demonstrating the lack of products in the formamide prior to the reaction.

Figure S.10. HR-direct MS results of water following heating in the presence of as-received catalyst particles. Each trace depicts the temporal profile of a specific mass of interest, demonstrating the purity of the catalyst's particles.

Figure S.11. HR-direct MS results of water following heating in the presence of as-received catalyst particles (continued). Each trace depicts the temporal profile of a specific mass of interest, demonstrating the purity of the catalyst's particles.

Figure S.122. HR-direct MS results of water following heating in the presence of as-received catalyst particles (continued). Each trace depicts the temporal profile of a specific mass of interest, demonstrating the purity of the catalyst particles.

3. LC-MS Results

3A.Calibration Curves (DNA/RNA building blocks) for LC-MS measurements

Figure S.213. Calibration graphs of commercial compounds used for products' quantification.

3B. LC-MS Results of Crude Product After Reaction

Figure S.14. LC-MS results of formamide and $CePO_4$ after 48 hours under 170°C and UV irradiation.

Figure S.25. LC-MS results of the product adenine after reaction of formamide and $CePO_4$ for 48 hours under 170°C and UV irradiation

Figure S.26. LC-MS results of the product cAMP after reaction of formamide and $CePO_4$ for 48 hours under 170°C and UV irradiation

Figure S.27. LC-MS results of the product CMP after reaction of formamide and $CePO_4$ for 48 hours under 170°C and UV irradiation

Figure S.28. LC-MS results of the product thymidine after reaction of formamide and $CePO_4$ for 48 hours under 170°C and UV irradiation

Figure S.29. LC-MS results of the product GMP after reaction of formamide and $CePO_4$ for 48 hours under 170°C and UV irradiation

Figure S.30. LC-MS results of the product cytosine after reaction of formamide and $CePO_4$ for 48 hours under 170°C and UV irradiation

Figure S.15. LC-MS results of the product thymine after reaction of formamide and $CePO_4$ for 48 hours under 170°C and UV irradiation

4. Adsorption experiments

4A.Calibration Curves

Figure S.32. Calibration curve for Adenine (based on absorption at 260.5nm).

Figure S.33. Calibration curve for Adenosine (based on absorption at 259.5 nm).

Figure S.34. Calibration curve for AMP (based on absorption at 259.5nm).

Figure S.35. Calibration curve for cAMP calibration (based on absorption at 260nm).

4B. Adsorption Kinetics of Adenine, Adenosine, AMP, and cAMP on the Catalyst Surface

Figure S.36. Adsorption kinetics of adenine on$CePO_4$ at 27°C.

Figure S.37. Adsorption kinetics of adenosine on $CePO_4$ at 27°C.

Figure S.38. Adsorption kinetics of AMP on$CePO_4$ at 27°C.

Figure S.16. Adsorption kinetics of cAMP on $CePO_4$ at 27°C.

5. Change in Turbidity During the Reaction

Figure S.40. The change in the absorption along the progression of the reaction in the presence of $CePO_4$ at 170°C and under UV-irradiation, in a duplicate (grey - time 0, green and brown 4h, yellow and cyan 7h, blue and orange 22 h).

Figure S.41. The change in the absorption along the progression of the reaction without catalyst at 170°C and under UV-irradiation, in duplicate (grey - time 0, green and brown 4h, yellow and cyan 7h, blue and orange 22h).

Figure S.42. The change in the absorption along the progression of the reaction formamide without catalyst at 170°C and in the dark, in duplicate (grey - time 0, green and brown 4h, yellow and cyan 7h, blue and orange 22h).

Figure S.43. The change in the absorption along the progression of the reaction formamide in the presence of $CePO_4$ at 170°C and in the dark, in duplicate (grey - time 0, green and brown 4h, yellow and cyan 7h, blue and orange 22h).

Figure S.44: A comparison between the LC-MS results of one of the samples (CePO4+ light + formamide) [A] and the LC-MS signal of the standards [B-F].

6. XPS Results

Figure S.45: XPS results and atomic percentage for: $CePO_4$ prior to reaction (reference), $CePO_4$ following reaction (and washing): after 48 hours under light and in the dark and (S2, S3 respectively) and after 24 hours under light and in the dark (S4, S5 respectively). In the inset table: the ratio between the atomic percentage of cerium to that of phosphorous in the various samples.

7. Additional Information

Figure S.46 The various RNA/DNA building blocks discussed in this manuscript

Figure S.47 All detected masses from a high-resolution direct-MS measurement of a sample taken following 48 hours of reaction of formamide at 170°C in the presence of $CePO_4$ and under UV-irradiation.

Figure S.48 The Total Ions Current (TIC) signal of the LC-MS following 48 hours of reaction of formamide at 170°C in the presence of $CePO_4$, under dark conditions.

Figure S.49 The Total Ions Current (TIC) signal of the LC-MS following 48 hours of reaction of formamide at 170°C in the presence of $CePO_4$ and under UV-irradiation.

List of Tables

1. **Catalyst Characterization - $CePO_4$**

Table S. 1. List of $CePO_4$ XRD peaks.

Table S. 2. BET - measurement of $CePO_4$

Table S. 3. Calculated Band Gap for $CePO_4$

4. **Adsorption experiments**

Table S. 4. The composition of each point in the calibration curve.

Table S.5. Calculated surface coverage in the adsorption measurements.

6. **XPS Results**

Table S.6: Atomic concentrations of O, C, P, Ce, N as obtained by XPS measurements prior to reaction (reference) and following reaction at various conditions. The ratios between the various elements are also given.

1. **<u>Catalyst Characterization - $CePO_4$</u>**

<u>XRD</u>

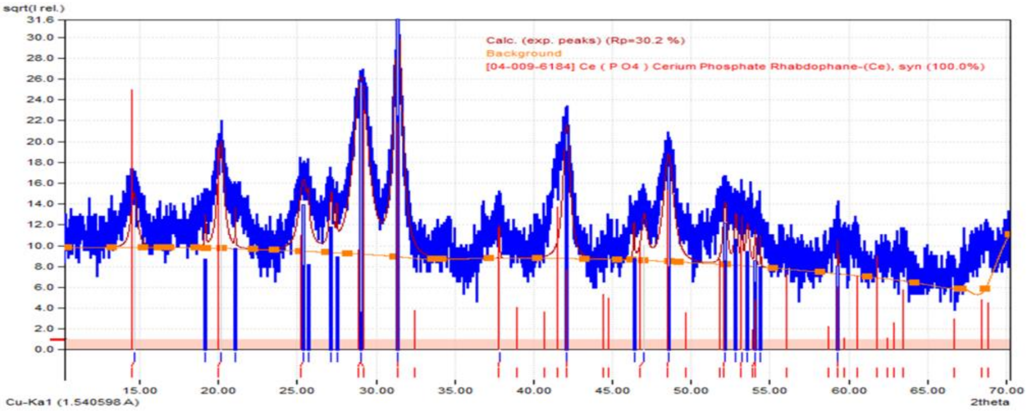

Figure S.17. XRD pattern of the catalyst $CePO_4$

Table S.5. List of $CePO_4$ XRD peaks.

No.	theta (rad)	2theta [º]	d [Å]	I/I0 (peak height)	Counts (peak area)	FWHM [rad]	FWHM [º]	Matched	Ţ(A)	Ţ(nm)
1	0.12776	14.64	6.0458	143.57	10.47	0.00733	0.42	A	190.629699	19.063
2	0.16729	19.17	4.6261	74.06	1.03	0.0014	0.08		1006.7033	100.67
3	0.17584	20.15	4.4033	335.88	24.48	0.00733	0.42	A	192.037365	19.2037
4	0.18387	21.07	4.2131	93.78	0.65	0.0007	0.04		2019.33793	201.934
5	0.22166	25.4	3.5038	192.22	20.69	0.01082	0.62	A	131.296008	13.1296
6	0.22436	25.71	3.4623	66.19	1.38	0.00209	0.12		678.779012	67.8779
7	0.23702	27.16	3.2806	137.4	6.2	0.00454	0.26		314.215083	31.4215
8	0.24007	27.51	3.2397	78.38	0.82	0.00105	0.06		1362.61037	136.261
9	0.25342	29.04	3.0724	658.22	102.82	0.01571	0.9	A	91.1467097	9.11467
10	0.27402	31.4	2.8466	1000	90.25	0.00908	0.52	A	158.633633	15.8634
11	0.33039	37.86	2.3744	72.19	0.5	0.0007	0.04	A	2098.81167	209.881
12	0.36739	42.1	2.1446	429.09	32.77	0.00768	0.44	A	193.386931	19.3387
13	0.40465	46.37	1.9566	76.42	1.06	0.0014	0.08		1079.86061	107.986
14	0.41041	47.03	1.9306	100.55	6.63	0.00663	0.38	A	227.905043	22.7905
15	0.42385	48.57	1.873	308.88	34.31	0.01117	0.64	A	136.126909	13.6127
16	0.45518	52.16	1.7522	146.87	7.14	0.00489	0.28	A	315.764952	31.5765
17	0.4612	52.85	1.7309	111.24	4.25	0.00384	0.22		403.077934	40.3078
18	0.46452	53.23	1.7194	95.29	1.32	0.0014	0.08	A	1110.30013	111.03
19	0.46757	53.58	1.709	87.8	3.96	0.00454	0.26		342.156072	34.2156
20	0.47185	54.07	1.6947	71.54	1.24	0.00175	0.1	A	891.538814	89.1539
21	0.47482	54.41	1.6849	62.57	0.43	0.0007	0.04		2232.23663	223.224
22	0.51775	59.33	1.5564	63.59	0.22	0.00035	0.02	A	4569.50409	456.95

The average size of the catalyst's particles was calculated using the Scherrer equation, upon averaging size calculated based on the three main peaks (9,10,12) in table S.1. The calculated average was 14.77nm with a standard deviation of 4.245.

Surface Area

Table S.6. BET – measurement of $CePO_4$

Sample	Weight (gr)	Heating time (hr.)	t° C	SA (m^2/gr)
$CePO_4$	0.1108	02:00	140	46.4

Energy Bandgap

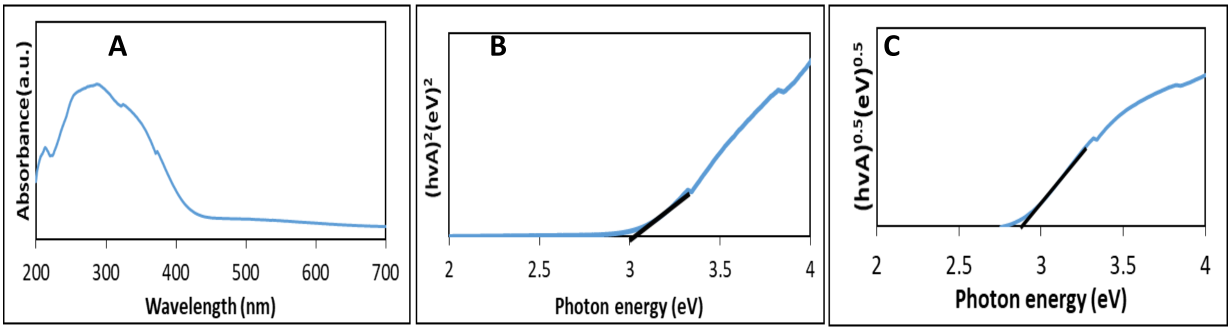

Figure S.18. (A) The UV-vis diffuse reflectance spectrum of $CePO_4$, (B) Tauc plot assuming indirect transition [$(hvA)^2$ VS. photon energy], (C) Tauc plot assuming direct transition [$(hvA)^{0.5}$ VS. photon energy]

Table S.7. Calculated Band Gap for $CePO_4$

The particle	Edge of absorbance	In - direct	Direct
$CePO_4$	436nm	3.00eV	2.90eV

2. Products Identification Using HR-direct MS

2A. HR-direct MS Results of Life-Building Blocks After the One-Pot Reaction of Formamide and $CePO_4$

The primely identification of the crude product was obtained by high resolution direct MS (in positive ionization +1). Figures S.3 – S.16) show the compounds identified by HR-MS after 48 hours of formamide reaction in the presence of $CePO_4$ at 170°C and under UV irradiation. The ion formula contains extra hydrogen (and +1 in the total m/z) since the measurements were performed in positive ionization. All results were

Compound Spectrum SmartFormula Report

Analysis Info

Analysis Name	D:\Data\Client\cli_3827 R1 direct_RA7_01_6347.d	Acquisition Date	01/02/2023 17:39:21
Method	direct pos new.m	Operator	Larisa Panz
Sample Name	cli_3827 R1 direct	Instrument	maXis impact 282001.00128
Comment			

Acquisition Parameter

Source Type	ESI	Ion Polarity	Positive	Set Nebulizer	3.0 Bar
Focus	Active	Set Capillary	4500 V	Set Dry Heater	180 °C
Scan Begin	50 m/z	Set End Plate Offset	-500 V	Set Dry Gas	8.0 l/min
Scan End	3000 m/z	Set Charging Voltage	2000 V	Set Divert Valve	Source
		Set Corona	0 nA	Set APCI Heater	0 °C

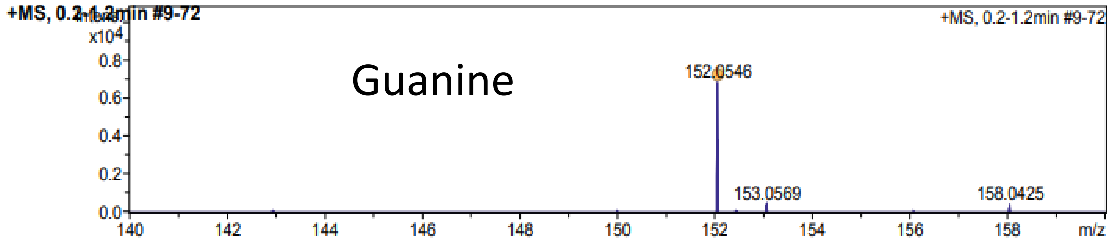

Meas. m/z	#	Ion Formula	m/z	err [ppm]	mSigma	# mSigma	Score	rdb	e¯ Conf	N-Rule	|err| [mDa]
152.0546	1	C5H6N5O	152.0572	17.3	6.2	1	100.00	5.5	even	ok	2.6
	1	C5H6N5O	152.0572	17.3	6.2	1	100.00	5.5	even	ok	2.6

Figure S.319. HR-direct MS mass results of the nucleobase guanine found in the reactor vessel following 48 hrs. of reaction at 170°C under light and in the presence of cerium phosphate.

Compound Spectrum SmartFormula Report

Analysis Info

Analysis Name	D:\Data\Client\cli_3827 R1 direct_RA7_01_6347.d	Acquisition Date	01/02/2023 17:39:21
Method	direct pos new.m	Operator	Larisa Panz
Sample Name	cli_3827 R1 direct	Instrument	maXis impact 282001.00128
Comment			

Acquisition Parameter

Source Type	ESI	Ion Polarity	Positive	Set Nebulizer	3.0 Bar
Focus	Active	Set Capillary	4500 V	Set Dry Heater	180 °C
Scan Begin	50 m/z	Set End Plate Offset	-500 V	Set Dry Gas	8.0 l/min
Scan End	3000 m/z	Set Charging Voltage	2000 V	Set Divert Valve	Source
		Set Corona	0 nA	Set APCI Heater	0 °C

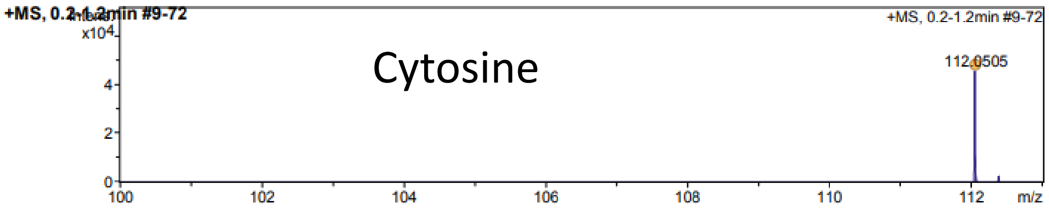

Meas. m/z	#	Ion Formula	m/z	err [ppm]	mSigma	# mSigma	Score	rdb	e⁻ Conf	N-Rule	|err| [mDa]	|err| [mDa]
112.0505	1	C4H6N3O	112.0511	5.3	4.6	1	100.00	3.5	even	ok	0.6	0.6
	1	C4H6N3O	112.0511	5.3	4.6	1	100.00	3.5	even	ok	0.6	0.6

Figure S.4. HR-direct MS results of the nucleobase cytosine found in the reactor vessel following 48 hrs. of reaction at 170°C under light and in the presence of cerium phosphate.

Compound Spectrum SmartFormula Report

Analysis Info

Analysis Name	D:\Data\Client\cli_3827 R1 direct_RA7_01_6347.d	Acquisition Date	01/02/2023 17:39:21
Method	direct pos new.m	Operator	Larisa Panz
Sample Name	cli_3827 R1 direct	Instrument	maXis impact 282001.00128
Comment			

Acquisition Parameter

Source Type	ESI	Ion Polarity	Positive	Set Nebulizer	3.0 Bar
Focus	Active	Set Capillary	4500 V	Set Dry Heater	180 °C
Scan Begin	50 m/z	Set End Plate Offset	-500 V	Set Dry Gas	8.0 l/min
Scan End	3000 m/z	Set Charging Voltage	2000 V	Set Divert Valve	Source
		Set Corona	0 nA	Set APCI Heater	0 °C

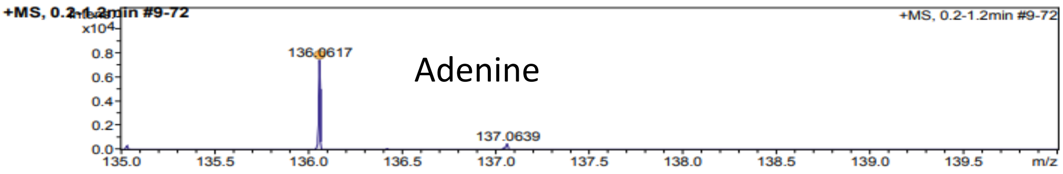

Meas. m/z	#	Ion Formula	m/z	err [ppm]	mSigma	# mSigma	Score	rdb	e⁻ Conf	N-Rule	|err| [mDa]	|err| [mDa]	|err| [mDa]
136.0617	1	C5H6N5	136.0623	4.3	6.4	1	100.00	5.5	even	ok	0.6	0.6	0.6
	1	C5H6N5	136.0623	4.3	6.4	1	100.00	5.5	even	ok	0.6	0.6	0.6

Figure S.5. HR-direct MS results of the nucleobase adenine found in the reactor vessel following 48 hrs. of reaction at 170°C under light and in the presence of cerium phosphate.

Compound Spectrum SmartFormula Report

**Analysis Info**		Acquisition Date	01/02/2023 17:39:21
Analysis Name	D:\Data\Client\cli_3827 R1 direct_RA7_01_6347.d		
Method	direct pos new.m	Operator	Larisa Panz
Sample Name	cli_3827 R1 direct	Instrument	maXis impact 282001.00128
Comment			

Acquisition Parameter

Source Type	ESI	Ion Polarity	Positive	Set Nebulizer	3.0 Bar
Focus	Active	Set Capillary	4500 V	Set Dry Heater	180 °C
Scan Begin	50 m/z	Set End Plate Offset	-500 V	Set Dry Gas	8.0 l/min
Scan End	3000 m/z	Set Charging Voltage	2000 V	Set Divert Valve	Source
		Set Corona	0 nA	Set APCI Heater	0 °C

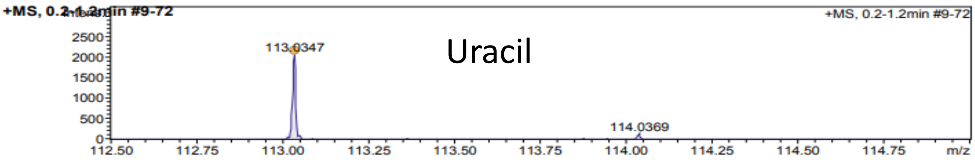

Meas. m/z	#	Ion Formula	m/z	err [ppm]	mSigma	# mSigma	Score	rdb	e¯ Conf	N-Rule	|err| [mDa]	|err| [mDa]
113.0347	1	C4H5N2O2	113.0351	3.1	11.3	1	100.00	3.5	even	ok	0.4	0.4
	1	C4H5N2O2	113.0351	3.1	11.3	1	100.00	3.5	even	ok	0.4	0.4

Figure S.6. HR-direct MS results of the nucleobase uracil found in the reactor vessel following 48 hrs. of reaction at 170°C under light and in the presence of cerium phosphate.

Compound Spectrum SmartFormula Report

**Analysis Info**		Acquisition Date	01/02/2023 17:39:21
Analysis Name	D:\Data\Client\cli_3827 R1 direct_RA7_01_6347.d		
Method	direct pos new.m	Operator	Larisa Panz
Sample Name	cli_3827 R1 direct	Instrument	maXis impact 282001.00128
Comment			

Acquisition Parameter

Source Type	ESI	Ion Polarity	Positive	Set Nebulizer	3.0 Bar
Focus	Active	Set Capillary	4500 V	Set Dry Heater	180 °C
Scan Begin	50 m/z	Set End Plate Offset	-500 V	Set Dry Gas	8.0 l/min
Scan End	3000 m/z	Set Charging Voltage	2000 V	Set Divert Valve	Source
		Set Corona	0 nA	Set APCI Heater	0 °C

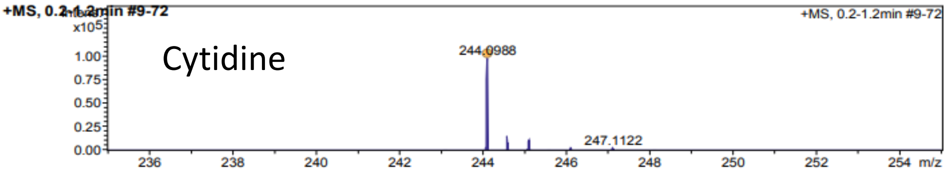

Meas. m/z	#	Ion Formula	m/z	err [ppm]	mSigma	# mSigma	Score	rdb	e¯ Conf	N-Rule	|err| [mDa]	|err| [mDa]	|err| [mDa]	|err| [mDa]	|err| [mDa]
244.0988	1	C9H14N3O5	244.0933	-22.2	1.7	1	100.00	4.5	even	ok	5.4	5.4	5.4	5.4	5.4
	1	C9H14N3O5	244.0933	-22.2	1.7	1	100.00	4.5	even	ok	5.4	5.4	5.4	5.4	5.4

Figure S.7. HR-direct MS results of the nucleoside cytidine found in the reactor vessel following 48 hrs. of reaction at 170°C under light and in the presence of cerium phosphate.

Compound Spectrum SmartFormula Report

Analysis Info

Analysis Name	D:\Data\Client\cli_3827 R1 direct_RA7_01_6347.d	Acquisition Date	01/02/2023 17:39:21
Method	direct pos new.m	Operator	Larisa Panz
Sample Name	cli_3827 R1 direct	Instrument	maXis impact 282001.00128
Comment			

Acquisition Parameter

Source Type	ESI	Ion Polarity	Positive	Set Nebulizer	3.0 Bar
Focus	Active	Set Capillary	4500 V	Set Dry Heater	180 °C
Scan Begin	50 m/z	Set End Plate Offset	-500 V	Set Dry Gas	8.0 l/min
Scan End	3000 m/z	Set Charging Voltage	2000 V	Set Divert Valve	Source
		Set Corona	0 nA	Set APCI Heater	0 °C

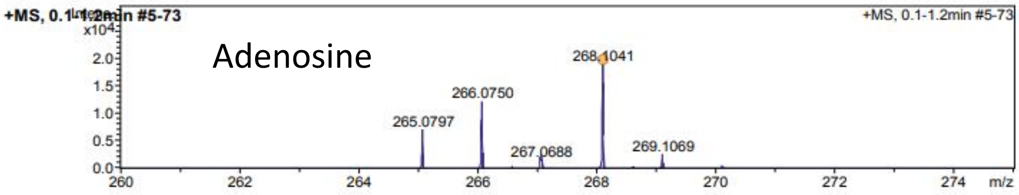

Meas. m/z	#	Ion Formula	m/z	err [ppm]	mSigma	# mSigma	Score	rdb	e⁻ Conf	N-Rule	|err| [mDa]	|err| [mDa]	|err| [mDa]	|err| [mDa]	|err| [mDa]
268.1041	1	C10H14N5O4	268.1046	1.9	2.9	1	100.00	6.5	even	ok	0.5	0.5	0.5	0.5	0.5
	1	C10H14N5O4	268.1046	1.9	2.9	1	100.00	6.5	even	ok	0.5	0.5	0.5	0.5	0.5

Figure S.8. HR-direct MS results of the nucleoside adenosine found in the reactor vessel following 48 hrs. of reaction at 170°C under light and in the presence of cerium phosphate.

Compound Spectrum SmartFormula Report

Analysis Info

Analysis Name	D:\Data\Client\cli_3827 R1 direct_RA7_01_6347.d	Acquisition Date	01/02/2023 17:39:21
Method	direct pos new.m	Operator	Larisa Panz
Sample Name	cli_3827 R1 direct	Instrument	maXis impact 282001.00128
Comment			

Acquisition Parameter

Source Type	ESI	Ion Polarity	Positive	Set Nebulizer	3.0 Bar
Focus	Active	Set Capillary	4500 V	Set Dry Heater	180 °C
Scan Begin	50 m/z	Set End Plate Offset	-500 V	Set Dry Gas	8.0 l/min
Scan End	3000 m/z	Set Charging Voltage	2000 V	Set Divert Valve	Source
		Set Corona	0 nA	Set APCI Heater	0 °C

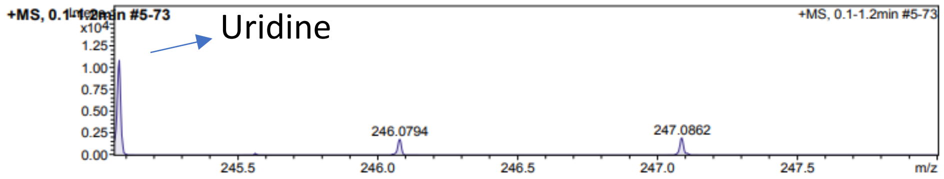

Meas. m/z	#	Ion Formula	m/z	err [ppm]	mSigma	# mSigma	Score	rdb	e⁻ Conf	N-Rule	|err| [mDa]	|err| [mDa]	|err| [mDa]	|err| [mDa]	|err| [mDa]
245.0770	1	C9H13N2O6	245.0774	1.4	101.5	1	100.00	4.5	even	ok	0.3	0.3	0.3	0.3	0.3
	1	C9H13N2O6	245.0774	1.4	101.5	1	100.00	4.5	even	ok	0.3	0.3	0.3	0.3	0.3

Figure S.9. HR-direct MS results of the nucleoside uridine found in the reactor vessel following 48 hrs. of reaction at 170°C under light and in the presence of cerium phosphate.

Compound Spectrum SmartFormula Report

**Analysis Info**			
Analysis Name	D:\Data\Client\cli_3827 R1 direct_RA7_01_6346.d	Acquisition Date	01/02/2023 17:32:07
Method	direct neg new.m	Operator	Larisa Panz
Sample Name	cli_3827 R1 direct	Instrument	maXis impact 282001.00128
Comment			

Acquisition Parameter

Source Type	ESI	Ion Polarity	Negative	Set Nebulizer	3.0 Bar
Focus	Active	Set Capillary	3900 V	Set Dry Heater	180 °C
Scan Begin	50 m/z	Set End Plate Offset	-500 V	Set Dry Gas	8.0 l/min
Scan End	3000 m/z	Set Charging Voltage	2000 V	Set Divert Valve	Source
		Set Corona	0 nA	Set APCI Heater	0 °C

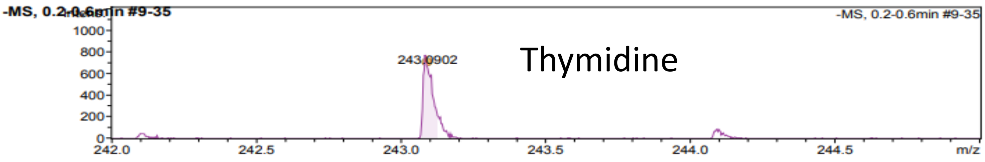

Meas. m/z	#	Ion Formula	m/z	err [ppm]	mSigma	# mSigma	Score	rdb	e⁻ Conf	N-Rule	|err| [mDa]
243.0902	1	C10H15N2O5	243.0981	32.3	n.a.	1	100.00	4.5	even	ok	7.9
	1	C10H15N2O5	243.0981	32.3	n.a.	1	100.00	4.5	even	ok	7.9

Figure S. 21. HR-direct MS results of the nucleoside thymidine found in the reactor vessel following 48 hrs. of reaction at 170°C under light and in the presence of cerium phosphate.

Compound Spectrum SmartFormula Report

**Analysis Info**			
Analysis Name	D:\Data\Client\cli_3827 R1 direct_RA7_01_6347.d	Acquisition Date	01/02/2023 17:39:21
Method	direct pos new.m	Operator	Larisa Panz
Sample Name	cli_3827 R1 direct	Instrument	maXis impact 282001.00128
Comment			

Acquisition Parameter

Source Type	ESI	Ion Polarity	Positive	Set Nebulizer	3.0 Bar
Focus	Active	Set Capillary	4500 V	Set Dry Heater	180 °C
Scan Begin	50 m/z	Set End Plate Offset	-500 V	Set Dry Gas	8.0 l/min
Scan End	3000 m/z	Set Charging Voltage	2000 V	Set Divert Valve	Source
		Set Corona	0 nA	Set APCI Heater	0 °C

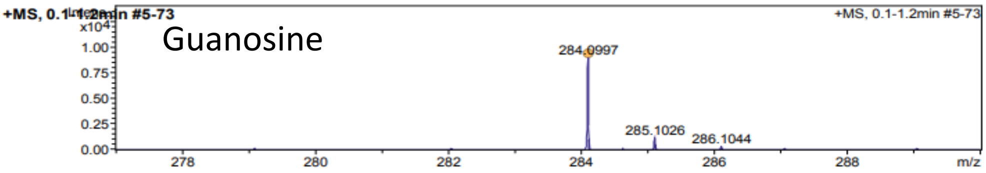

Meas. m/z	#	Ion Formula	m/z	err [ppm]	mSigma	# mSigma	Score	rdb	e⁻ Conf	N-Rule	|err| [mDa]	|err| [mDa]	|err| [mDa]	|err| [mDa]	|err| [mDa]
284.0997	1	C10H14N5O5	284.0995	-0.8	10.0	1	100.00	6.5	even	ok	0.2	0.2	0.2	0.2	0.2
	1	C10H14N5O5	284.0995	-0.8	10.0	1	100.00	6.5	even	ok	0.2	0.2	0.2	0.2	0.2

Figure S.20. HR-direct MS results of the nucleoside guanosine found in the reactor vessel following 48 hrs. of reaction at 170°C under light and in the presence of cerium phosphate.

Compound Spectrum SmartFormula Report

Analysis Info

Analysis Name	D:\Data\Client\cli_3827 R1 direct_RA7_01_6347.d	Acquisition Date	01/02/2023 17:39:21
Method	direct pos new.m	Operator	Larisa Panz
Sample Name	cli_3827 R1 direct	Instrument	maXis impact 282001.00128
Comment			

Acquisition Parameter

Source Type	ESI	Ion Polarity	Positive	Set Nebulizer	3.0 Bar
Focus	Active	Set Capillary	4500 V	Set Dry Heater	180 °C
Scan Begin	50 m/z	Set End Plate Offset	-500 V	Set Dry Gas	8.0 l/min
Scan End	3000 m/z	Set Charging Voltage	2000 V	Set Divert Valve	Source
		Set Corona	0 nA	Set APCI Heater	0 °C

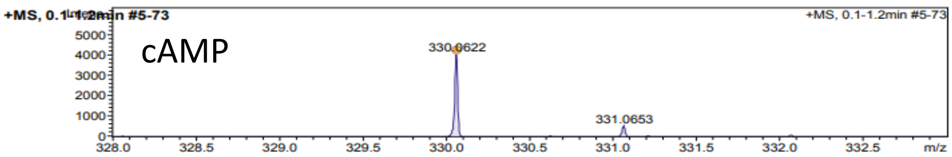

Meas. m/z	#	Ion Formula	m/z	err [ppm]	mSigma	# mSigma	Score	rdb	e⁻ Conf	N-Rule	|err| [mDa]	|err| [mDa]	|err| [mDa]	|err| [mDa]	|err| [mDa]
330.0622	1	C10H13N5O6P	330.0603	-5.7	11.7	1	100.00	7.5	even	ok	1.9	1.9	1.9	1.9	1.9
	1	C10H13N5O6P	330.0603	-5.7	11.7	1	100.00	7.5	even	ok	1.9	1.9	1.9	1.9	1.9

Figure S.23. HR-direct MS results of the cyclic nucleotide cAMP found in the reactor vessel following 48 hrs. of reaction at 170°C under light and in the presence of cerium phosphate.

Compound Spectrum SmartFormula Report

Analysis Info

Analysis Name	D:\Data\Client\cli_3827 R1 direct_RA7_01_6347.d	Acquisition Date	01/02/2023 17:39:21
Method	direct pos new.m	Operator	Larisa Panz
Sample Name	cli_3827 R1 direct	Instrument	maXis impact 282001.00128
Comment			

Acquisition Parameter

Source Type	ESI	Ion Polarity	Positive	Set Nebulizer	3.0 Bar
Focus	Active	Set Capillary	4500 V	Set Dry Heater	180 °C
Scan Begin	50 m/z	Set End Plate Offset	-500 V	Set Dry Gas	8.0 l/min
Scan End	3000 m/z	Set Charging Voltage	2000 V	Set Divert Valve	Source
		Set Corona	0 nA	Set APCI Heater	0 °C

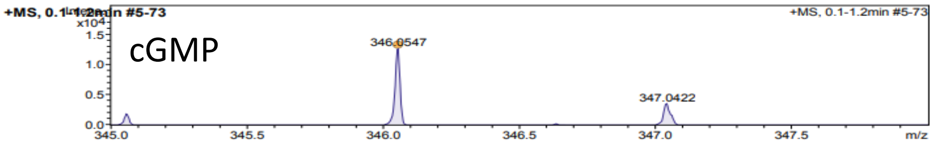

Meas. m/z	#	Ion Formula	m/z	err [ppm]	mSigma	# mSigma	Score	rdb	e⁻ Conf	N-Rule	|err| [mDa]	|err| [mDa]	|err| [mDa]	|err| [mDa]	|err| [mDa]
346.0547	1	C10H13N5O7P	346.0553	1.7	604.7	1	100.00	7.5	even	ok	0.6	0.6	0.6	0.6	0.6
	1	C10H13N5O7P	346.0553	1.7	604.7	1	100.00	7.5	even	ok	0.6	0.6	0.6	0.6	0.6

Figure S.22. HR-direct MS results of the cyclic nucleotide cGMP found in the reactor vessel following 48 hrs. of reaction at 170°C under light and in the presence of cerium phosphate.

Compound Spectrum SmartFormula Report

Analysis Info

Analysis Name	D:\Data\Client\cli_3827 R1 direct_RA7_01_6347.d	Acquisition Date	01/02/2023 17:39:21
Method	direct pos new.m	Operator	Larisa Panz
Sample Name	cli_3827 R1 direct	Instrument	maXis impact 282001.00128
Comment			

Acquisition Parameter

Source Type	ESI	Ion Polarity	Positive	Set Nebulizer	3.0 Bar
Focus	Active	Set Capillary	4500 V	Set Dry Heater	180 °C
Scan Begin	50 m/z	Set End Plate Offset	-500 V	Set Dry Gas	8.0 l/min
Scan End	3000 m/z	Set Charging Voltage	2000 V	Set Divert Valve	Source
		Set Corona	0 nA	Set APCI Heater	0 °C

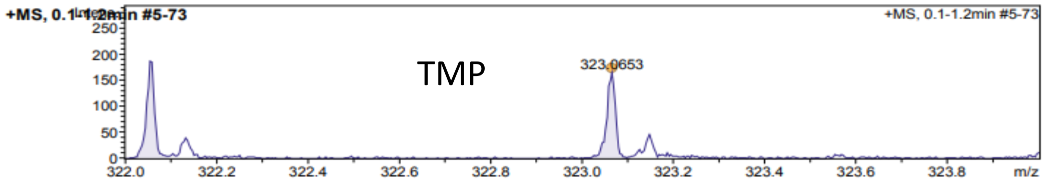

Meas. m/z	#	Ion Formula	m/z	err [ppm]	mSigma	# mSigma	Score	rdb	e⁻ Conf	N-Rule	|err| [mDa]	|err| [mDa]	|err| [mDa]	|err| [mDa]	|err| [mDa]
323.0653	1	C10H16N2O8P	323.0644	-2.7	763.6	1	100.00	4.5	even	ok	0.9	0.9	0.9	0.9	0.9
	1	C10H16N2O8P	323.0644	-2.7	763.6	1	100.00	4.5	even	ok	0.9	0.9	0.9	0.9	0.9

Figure S.14. HR-direct MS results of the nucleotide TMP found in the reactor vessel following 48 hrs. of reaction at 170°C under light and in the presence of cerium phosphate.

Compound Spectrum SmartFormula Report

Analysis Info

Analysis Name	D:\Data\Client\cli_3827 R1 direct_RA7_01_6347.d	Acquisition Date	01/02/2023 17:39:21
Method	direct pos new.m	Operator	Larisa Panz
Sample Name	cli_3827 R1 direct	Instrument	maXis impact 282001.00128
Comment			

Acquisition Parameter

Source Type	ESI	Ion Polarity	Positive	Set Nebulizer	3.0 Bar
Focus	Active	Set Capillary	4500 V	Set Dry Heater	180 °C
Scan Begin	50 m/z	Set End Plate Offset	-500 V	Set Dry Gas	8.0 l/min
Scan End	3000 m/z	Set Charging Voltage	2000 V	Set Divert Valve	Source
		Set Corona	0 nA	Set APCI Heater	0 °C

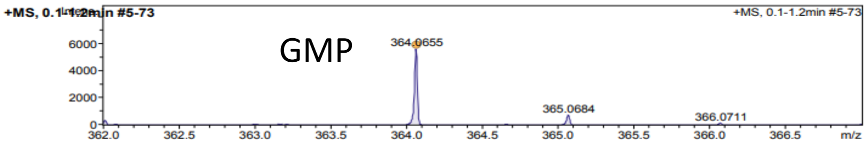

Meas. m/z	#	Ion Formula	m/z	err [ppm]	mSigma	# mSigma	Score	rdb	e⁻ Conf	N-Rule	|err| [mDa]	|err| [mDa]	|err| [mDa]	|err| [mDa]	|err| [mDa]
364.0655	1	C10H15N5O8P	364.0658	0.8	1.7	1	100.00	6.5	even	ok	0.3	0.3	0.3	0.3	0.3
	1	C10H15N5O8P	364.0658	0.8	1.7	1	100.00	6.5	even	ok	0.3	0.3	0.3	0.3	0.3

Figure S.25. HR-direct MS results of the nucleotide GMP found in the reactor vessel following 48 hrs. of reaction at 170°C under light and in the presence of cerium phosphate.

Compound Spectrum SmartFormula Report

Analysis Info

Analysis Name	D:\Data\Client\cli_3827 R1 direct_RA7_01_6347.d	Acquisition Date	01/02/2023 17:39:21
Method	direct pos new.m	Operator	Larisa Panz
Sample Name	cli_3827 R1 direct	Instrument	maXis impact 282001.00128
Comment			

Acquisition Parameter

Source Type	ESI	Ion Polarity	Positive	Set Nebulizer	3.0 Bar
Focus	Active	Set Capillary	4500 V	Set Dry Heater	180 °C
Scan Begin	50 m/z	Set End Plate Offset	-500 V	Set Dry Gas	8.0 l/min
Scan End	3000 m/z	Set Charging Voltage	2000 V	Set Divert Valve	Source
		Set Corona	0 nA	Set APCI Heater	0 °C

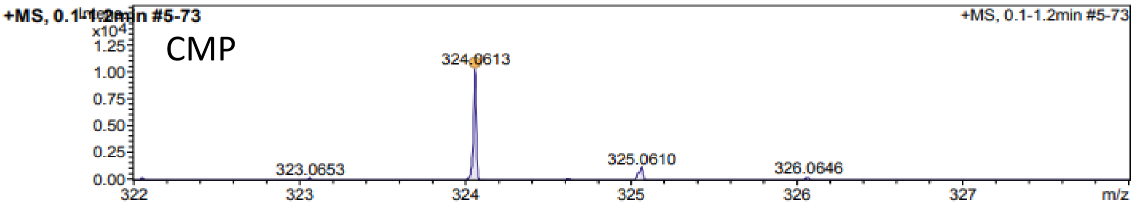

Meas. m/z	#	Ion Formula	m/z	err [ppm]	mSigma	# mSigma	Score	rdb	e⁻ Conf	N-Rule	|err| [mDa]	|err| [mDa]	|err| [mDa]	|err| [mDa]	|err| [mDa]
324.0613	1	C9H15N3O8P	324.0597	-4.9	2.9	1	100.00	4.5	even	ok	1.6	1.6	1.6	1.6	1.6
	1	C9H15N3O8P	324.0597	-4.9	2.9	1	100.00	4.5	even	ok	1.6	1.6	1.6	1.6	1.6

Figure S.26.HR-direct MS results of the nucleotide CMP found in the reactor vessel following 48 hrs. of reaction at 170°C under light and in the presence of cerium phosphate.

2B. HR-direct MS Results of Formamide and Water Following Heating in the presence of $CePO_4$

As part of the control experiments, formamide was measured by HR direct-MS prior to reaction to verify its purity and to assure that the products were formed during the reaction. In this measurement, the formamide was injected to the HR direct-MS and the molecular mass of each product was searched. As expected, no compounds were found (Figures S.17-S.19).

A second set of measurements was aimed at showing that the catalyst used for the reaction was obtained without any products adsorbed on its surface. For that, the catalyst's particles were introduced into a tube containing HPLC – grade water and the tube was heated to 90°C for one hour. Then the liquid was separated from the catalyst by centrifugation at 14,000 rpm for 10 min and measured by direct MS. Here, again, no compounds of interest could be observed (Figures S.20- S.22).

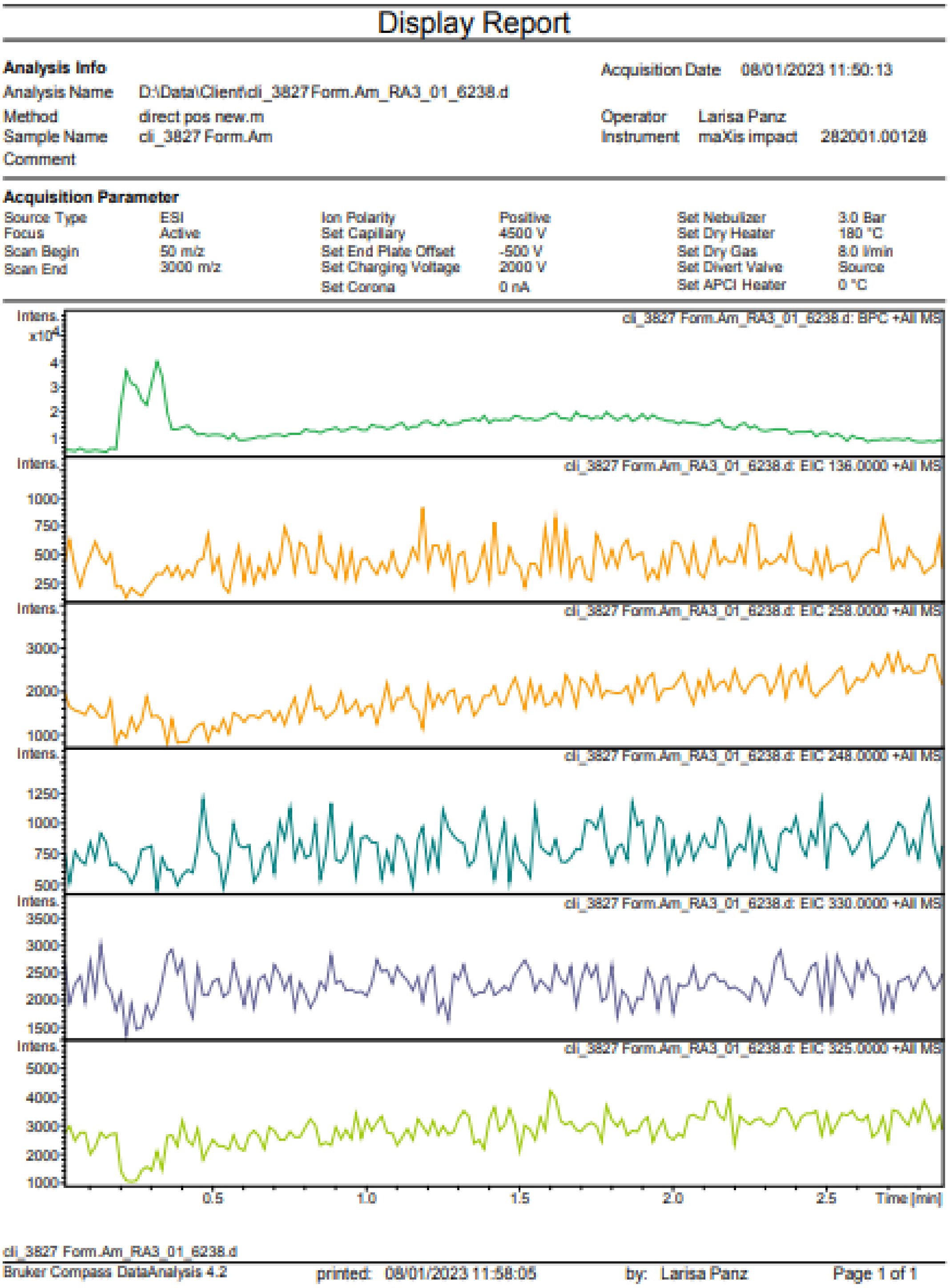

Figure S.27. HR-direct MS results of formamide as received. Each trace depicts the temporal profile of a specific mass of interest, demonstrating the lack of products in the formamide prior to reaction.

The upper trace presents the UV chromatogram as a function of time, measured at 260 nm. The presented signal intensities in all other traces represent the integrated signals between (m-0.5)/z and (m+0.5)/z, in which "m" is the nominal mass depicted in the legend.

Display Report

Analysis Info

Analysis Name	D:\Data\Client\cli_3827 Form.Am_RA3_01_6238.d	Acquisition Date	08/01/2023 11:50:13
Method	direct pos new.m	Operator	Larisa Panz
Sample Name	cli_3827 Form.Am	Instrument	maXis impact 282001.00128
Comment			

Acquisition Parameter

Source Type	ESI	Ion Polarity	Positive	Set Nebulizer	3.0 Bar
Focus	Active	Set Capillary	4500 V	Set Dry Heater	180 °C
Scan Begin	50 m/z	Set End Plate Offset	-500 V	Set Dry Gas	8.0 l/min
Scan End	3000 m/z	Set Charging Voltage	2000 V	Set Divert Valve	Source
		Set Corona	0 nA	Set APCI Heater	0 °C

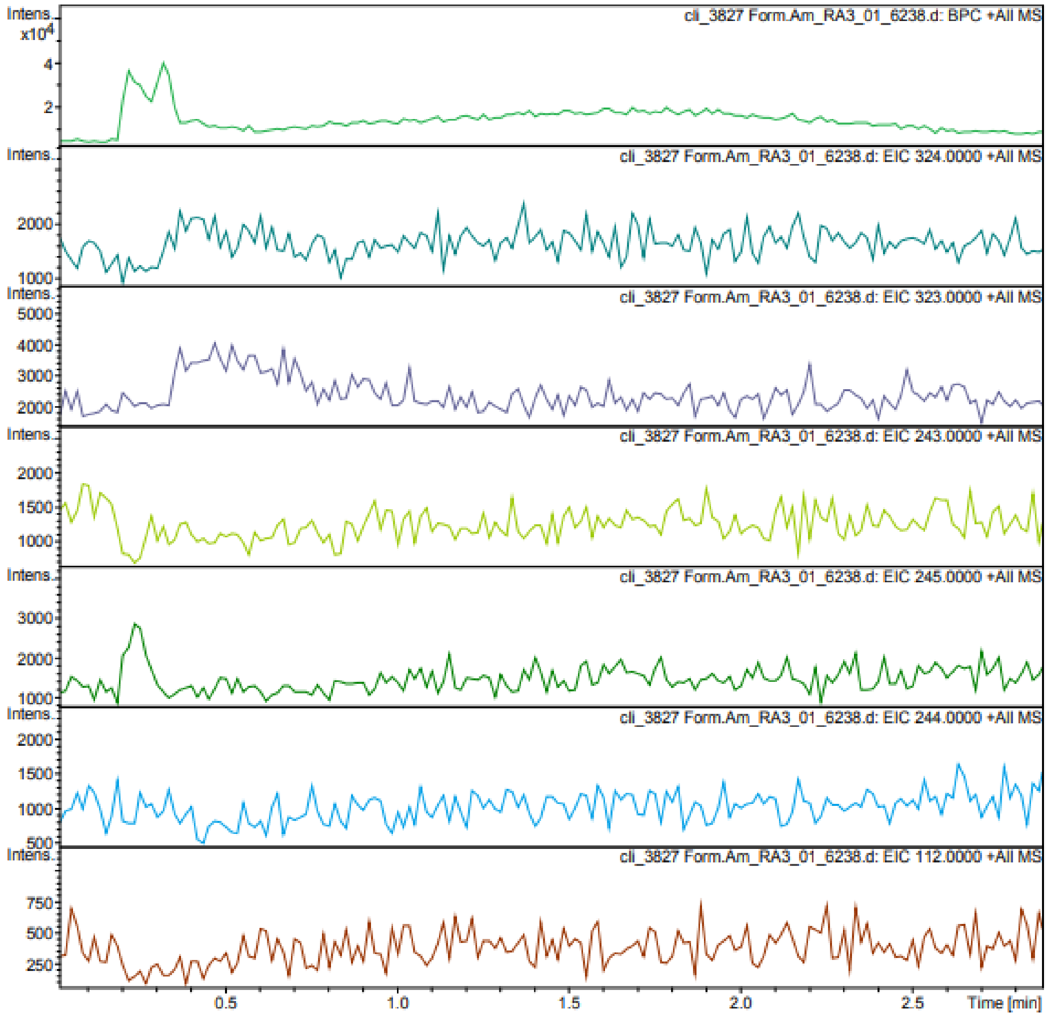

cli_3827 Form.Am_RA3_01_6238.d

Bruker Compass DataAnalysis 4.2 printed: 08/01/2023 12:02:46 by: Larisa Panz Page 1 of 1

Figure S.28. HR-direct MS results of formamide (continued). Each trace depicts the temporal profile of a specific mass of interest, demonstrating the lack of products in the formamide prior to reaction.

The upper trace presents the UV chromatogram as a function of time, measured at 260 nm. The presented signal intensities in the other traces represent the integrated signals between (m-0.5)/z and (m+0.5)/z, in which "m" is the nominal mass depicted in the legend.

Display Report

Analysis Info

Analysis Name	D:\Data\Client\cli_3827 Form.Am_RA3_01_6238.d	Acquisition Date	08/01/2023 11:50:13
Method	direct pos new.m	Operator	Larisa Panz
Sample Name	cli_3827 Form.Am	Instrument	maXis impact 282001.00128
Comment			

Acquisition Parameter

Source Type	ESI	Ion Polarity	Positive	Set Nebulizer	3.0 Bar
Focus	Active	Set Capillary	4500 V	Set Dry Heater	180 °C
Scan Begin	50 m/z	Set End Plate Offset	-500 V	Set Dry Gas	8.0 l/min
Scan End	3000 m/z	Set Charging Voltage	2000 V	Set Divert Valve	Source
		Set Corona	0 nA	Set APCI Heater	0 °C

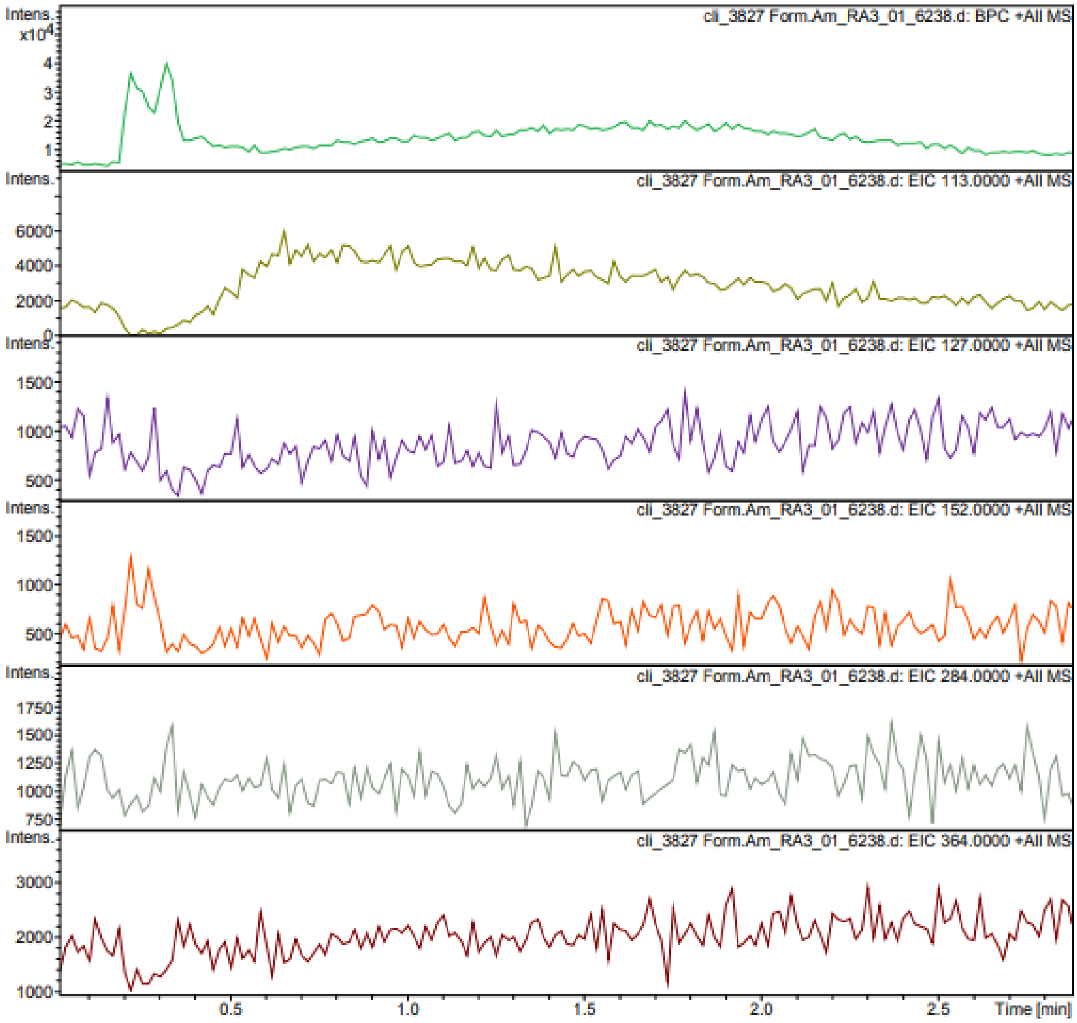

cli_3827 Form.Am_RA3_01_6238.d

Bruker Compass DataAnalysis 4.2 printed: 08/01/2023 12:04:25 by: Larisa Panz Page 1 of 1

Figure S.19. HR-direct MS results of formamide (continued). Each trace depicts the temporal profile of a specific mass of interest, demonstrating the lack of products in the formamide prior to reaction.

The upper trace presents the UV chromatogram as a function of time, measured at 260 nm. The presented signal intensities in all other traces represent the integrated signals between (m-0.5)/z and (m+0.5)/z, in which "m" is the nominal mass depicted in the legend.

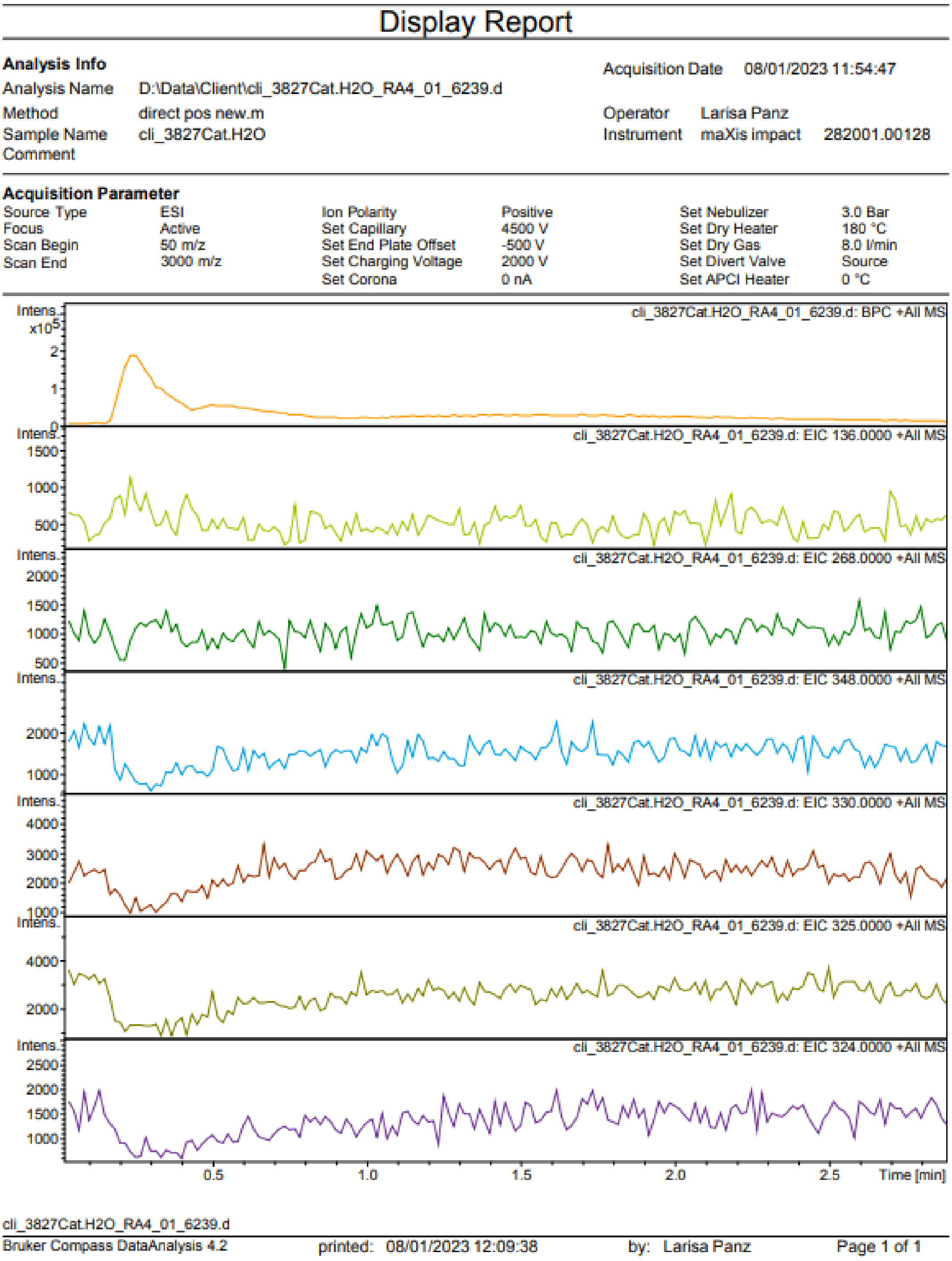

Figure S.29. HR-direct MS results of water following heating in the presence of as-received catalyst particles. Each trace depicts the temporal profile of a specific mass of interest, demonstrating the purity of the catalyst's particles.

The upper trace presents the UV chromatogram as a function of time, measured at 260 nm. The presented signal intensities in all other traces represent the integrated signals between (m-0.5)/z and (m+0.5)/z, in which "m" is the nominal mass depicted in the legend.

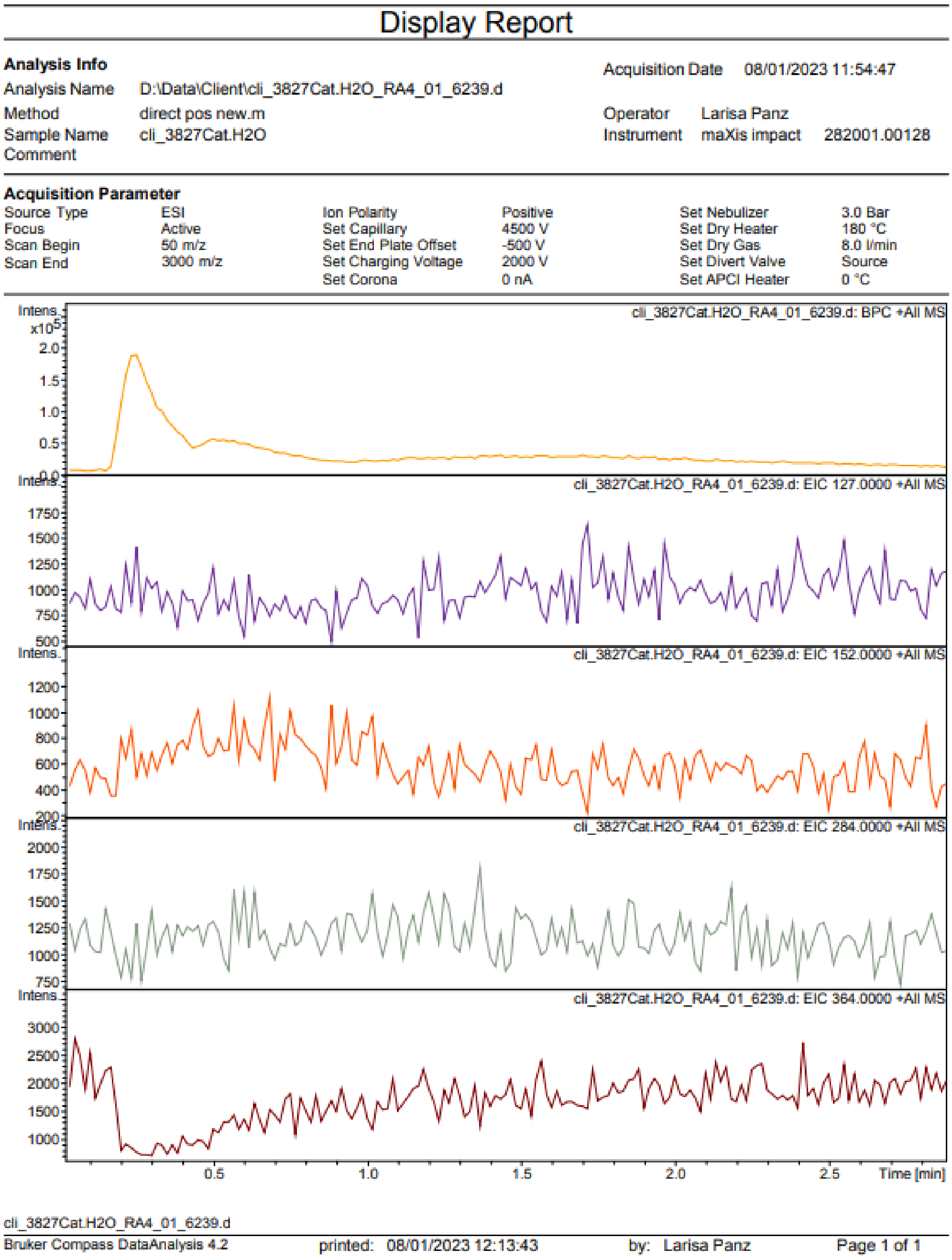
Display Report

Analysis Info

Analysis Name D:\Data\Client\cli_3827Cat.H2O_RA4_01_6239.d
Method direct pos new.m
Sample Name cli_3827Cat.H2O
Comment

Acquisition Date 08/01/2023 11:54:47
Operator Larisa Panz
Instrument maXis impact 282001.00128

Acquisition Parameter

Source Type	ESI	Ion Polarity	Positive	Set Nebulizer	3.0 Bar
Focus	Active	Set Capillary	4500 V	Set Dry Heater	180 °C
Scan Begin	50 m/z	Set End Plate Offset	-500 V	Set Dry Gas	8.0 l/min
Scan End	3000 m/z	Set Charging Voltage	2000 V	Set Divert Valve	Source
		Set Corona	0 nA	Set APCI Heater	0 °C

cli_3827Cat.H2O_RA4_01_6239.d
Bruker Compass DataAnalysis 4.2 printed: 08/01/2023 12:13:43 by: Larisa Panz Page 1 of 1

Figure S.30. HR-direct MS results of water following heating in the presence of as-received catalyst particles (continued). Each trace depicts the temporal profile of a specific mass of interest, demonstrating the purity of the catalyst's particles.

The upper trace presents the UV chromatogram as a function of time, measured at 260 nm The presented signal intensities in all other traces represent the integrated signals between (m-0.5)/z and (m+0.5)/z, in which "m" is the nominal mass depicted in the legend.

Display Report

Analysis Info

Analysis Name	D:\Data\Client\cli_3827Cat.H2O_RA4_01_6239.d	Acquisition Date	08/01/2023 11:54:47
Method	direct pos new.m	Operator	Larisa Panz
Sample Name	cli_3827Cat.H2O	Instrument	maXis impact 282001.00128
Comment			

Acquisition Parameter

Source Type	ESI	Ion Polarity	Positive	Set Nebulizer	3.0 Bar
Focus	Active	Set Capillary	4500 V	Set Dry Heater	180 °C
Scan Begin	50 m/z	Set End Plate Offset	-500 V	Set Dry Gas	8.0 l/min
Scan End	3000 m/z	Set Charging Voltage	2000 V	Set Divert Valve	Source
		Set Corona	0 nA	Set APCI Heater	0 °C

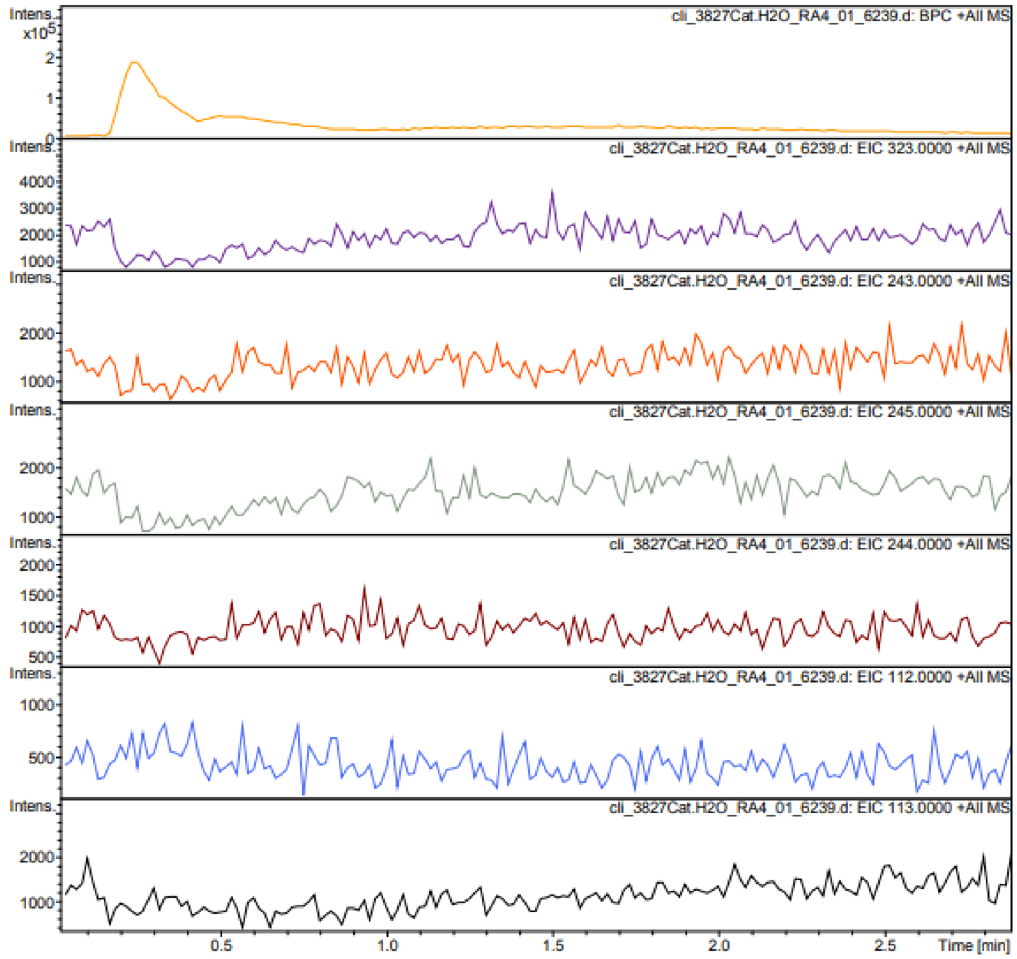

cli_3827Cat.H2O_RA4_01_6239.d

Bruker Compass DataAnalysis 4.2 printed: 08/01/2023 12:11:37 by: Larisa Panz Page 1 of 1

Figure S.31. HR-direct MS results of water following heating in the presence of as-received catalyst particles (continued). Each trace depicts the temporal profile of a specific mass of interest, demonstrating the purity of the catalyst particles.

The upper trace presents the UV chromatogram as a function of time, measured at 260 nm. The presented signal intensities in all other traces represent the integrated signals between (m-0.5)/z and (m+0.5)/z, in which “m” is the nominal mass depicted in the legend.

3. LC-MS Results

3A.Calibration Curves (DNA/RNA building blocks) for LC-MS measurements

Adenine		
conc. mM	intensity	/10^6
0.3	378481	0.378481
0.08	186064	0.186064
0.02	122624	0.122624
0.005	49660	0.04966

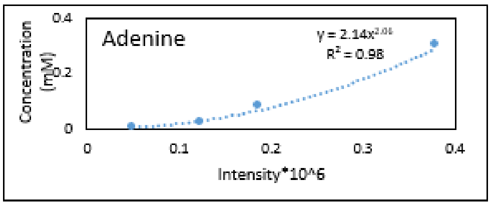

cAMP		
conc. mM	intensity	/10^6
0.08	583128	0.583128
0.02	171562	0.171562
0.005	33619	0.033619
0.002	13027	0.013027

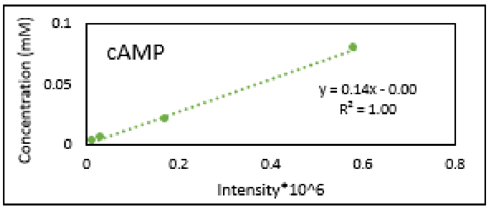

TMP		
conc. mM	intensity	/10^6
0.3	868188	0.868188
0.08	150824	0.150824
0.02	50414	0.050414
0.005	16948	0.016948

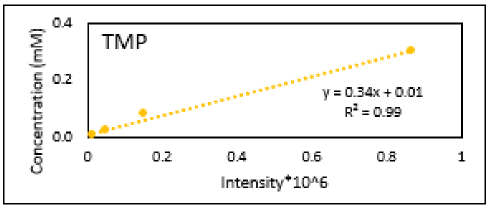

Thymidine		
conc. mM	intensity	/10^6
0.3	203102	0.203102
0.08	66019	0.066019
0.02	14500.3	0.0145
0.005	5786.4	0.005786
0.002	3284	0.003284

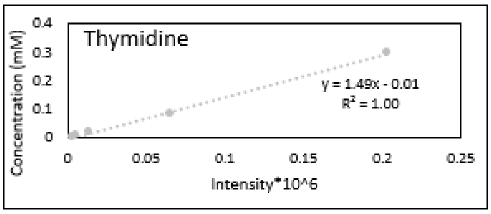

Thymine		
conc. mM	intensity	/10^6
0.3	459023	0.459023
0.08	99843	0.099843
0.02	36561	0.036561
0.005	8765	0.008765

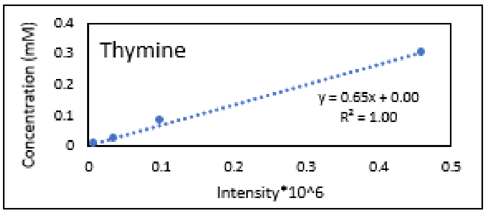

Uridine		
conc. mM	intensity	/10^6
0.6	4790335	4.790335
0.3	2258398	2.258398
0.08	1255025	1.255025
0.02	358575	0.358575
0.005	47767	0.047767

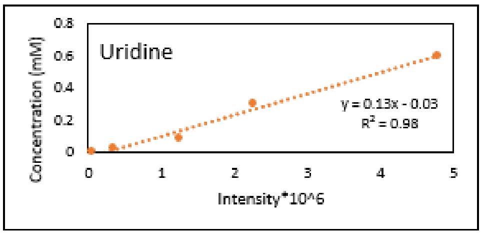

3B. LC-MS Results of Crude Product After Reaction

The identification and quantification of the crude product was obtained by high resolution LC-MS (in positive ionization +1). All result compered to commercial standards (part 3A).

Analysis Info

Analysis Name	D:\Data\Client\cli_3827sample1_RB2_01_6251.d	Acquisition Date	08/01/2023 18:47:57
Method	cli_3827.m	Operator	Larisa Panz
Sample Name	cli_3827 sample1	Instrument	maXis impact 282001.00128
Comment			

Acquisition Parameter

Source Type	ESI	Ion Polarity	Positive	Set Nebulizer	3.0 Bar
Focus	Active	Set Capillary	4500 V	Set Dry Heater	180 °C
Scan Begin	50 m/z	Set End Plate Offset	-500 V	Set Dry Gas	8.0 l/min
Scan End	3000 m/z	Set Charging Voltage	2000 V	Set Divert Valve	Source
		Set Corona	0 nA	Set APCI Heater	0 °C

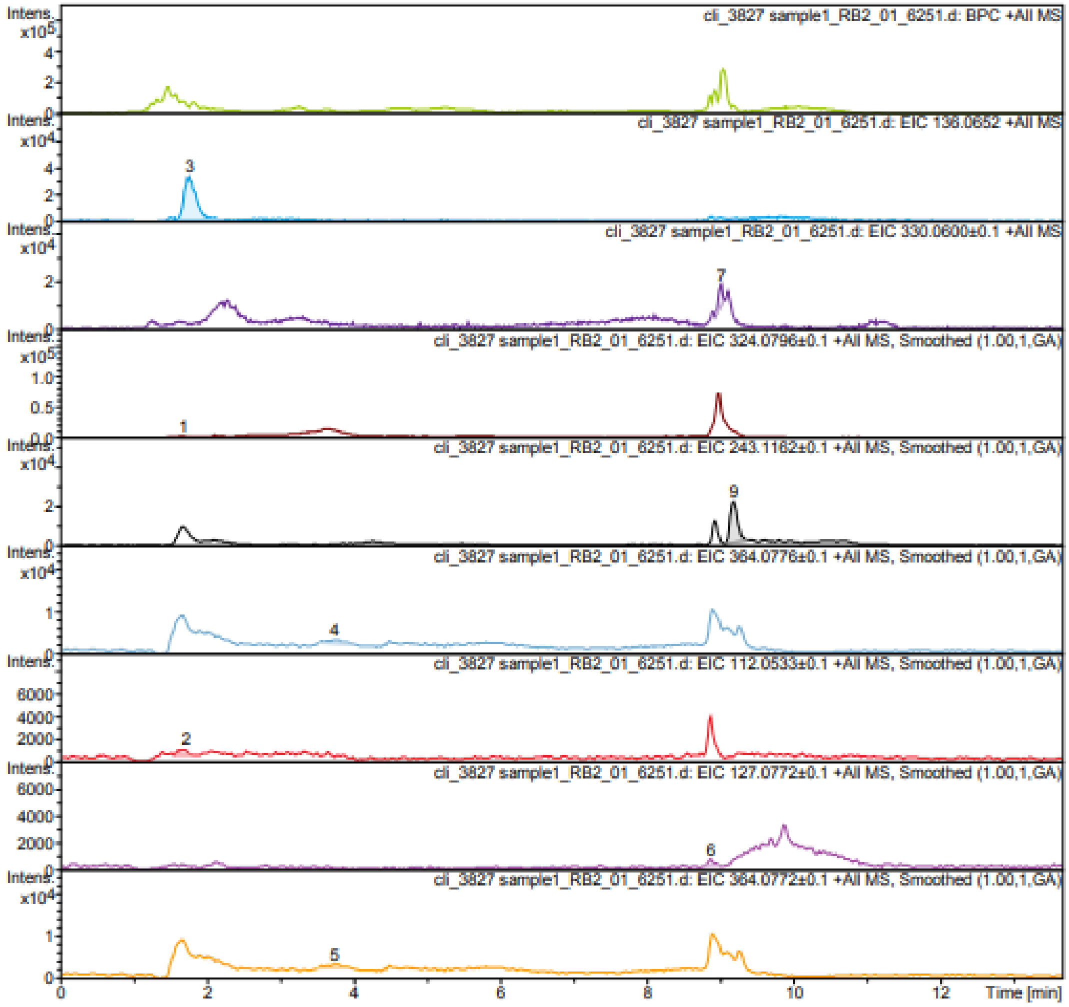

Figure S.32. LC-MS results of formamide and $CePO_4$ after 48 hours under 170°C and UV irradiation. The upper trace presents the UV chromatogram as a function of time, measured at 260 nm.

Display Report

Analysis Info

Analysis Name	D:\Data\Client\cli_3827 sample1_RB2_01_6251.d	Acquisition Date	08/01/2023 18:47:57
Method	cli_3827.m	Operator	Larisa Panz
Sample Name	cli_3827 sample1	Instrument	maXis impact 282001.00128
Comment			

Acquisition Parameter

Source Type	ESI	Ion Polarity	Positive	Set Nebulizer	3.0 Bar
Focus	Active	Set Capillary	4500 V	Set Dry Heater	180 °C
Scan Begin	50 m/z	Set End Plate Offset	-500 V	Set Dry Gas	8.0 l/min
Scan End	3000 m/z	Set Charging Voltage	2000 V	Set Divert Valve	Source
		Set Corona	0 nA	Set APCI Heater	0 °C

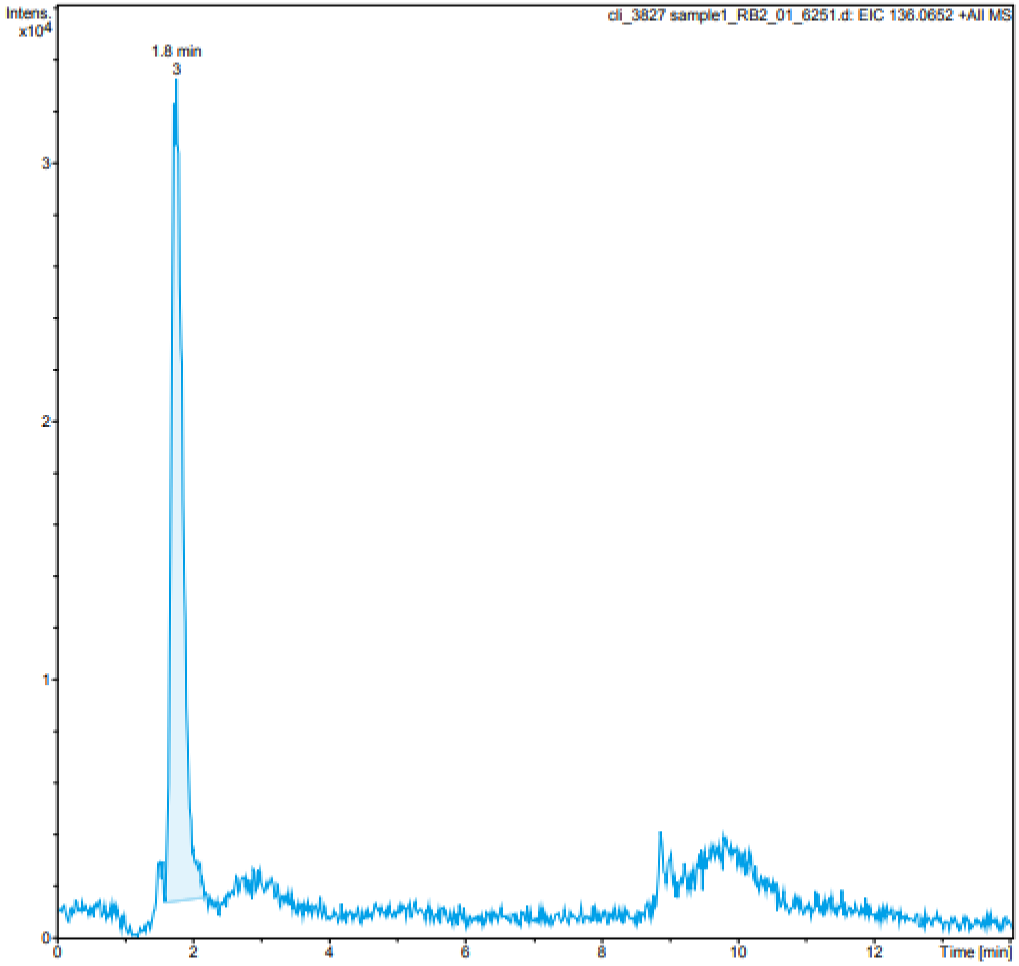

Figure S.25. LC-MS results of the product adenine after reaction of formamide and $CePO_4$ for 48 hours under 170°C and UV irradiation

Display Report

Analysis Info

Analysis Name	D:\Data\Client\cli_3827 sample1_RB2_01_6251.d	Acquisition Date	08/01/2023 18:47:57
Method	cli_3827.m	Operator	Larisa Panz
Sample Name	cli_3827 sample1	Instrument	maXis impact 282001.00128
Comment			

Acquisition Parameter

Source Type	ESI	Ion Polarity	Positive	Set Nebulizer	3.0 Bar
Focus	Active	Set Capillary	4500 V	Set Dry Heater	180 °C
Scan Begin	50 m/z	Set End Plate Offset	-500 V	Set Dry Gas	8.0 l/min
Scan End	3000 m/z	Set Charging Voltage	2000 V	Set Divert Valve	Source
		Set Corona	0 nA	Set APCI Heater	0 °C

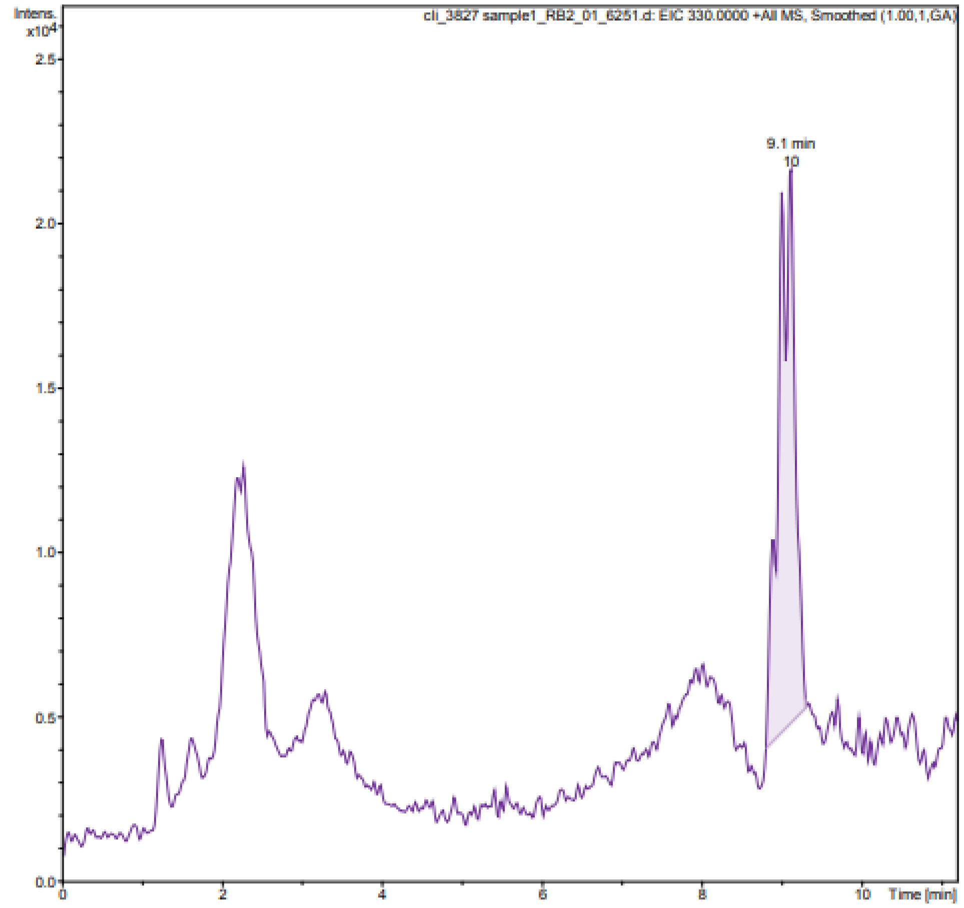

Figure S.26. LC-MS results of the product cAMP after reaction of formamide and $CePO_4$ for 48 hours under 170°C and UV irradiation

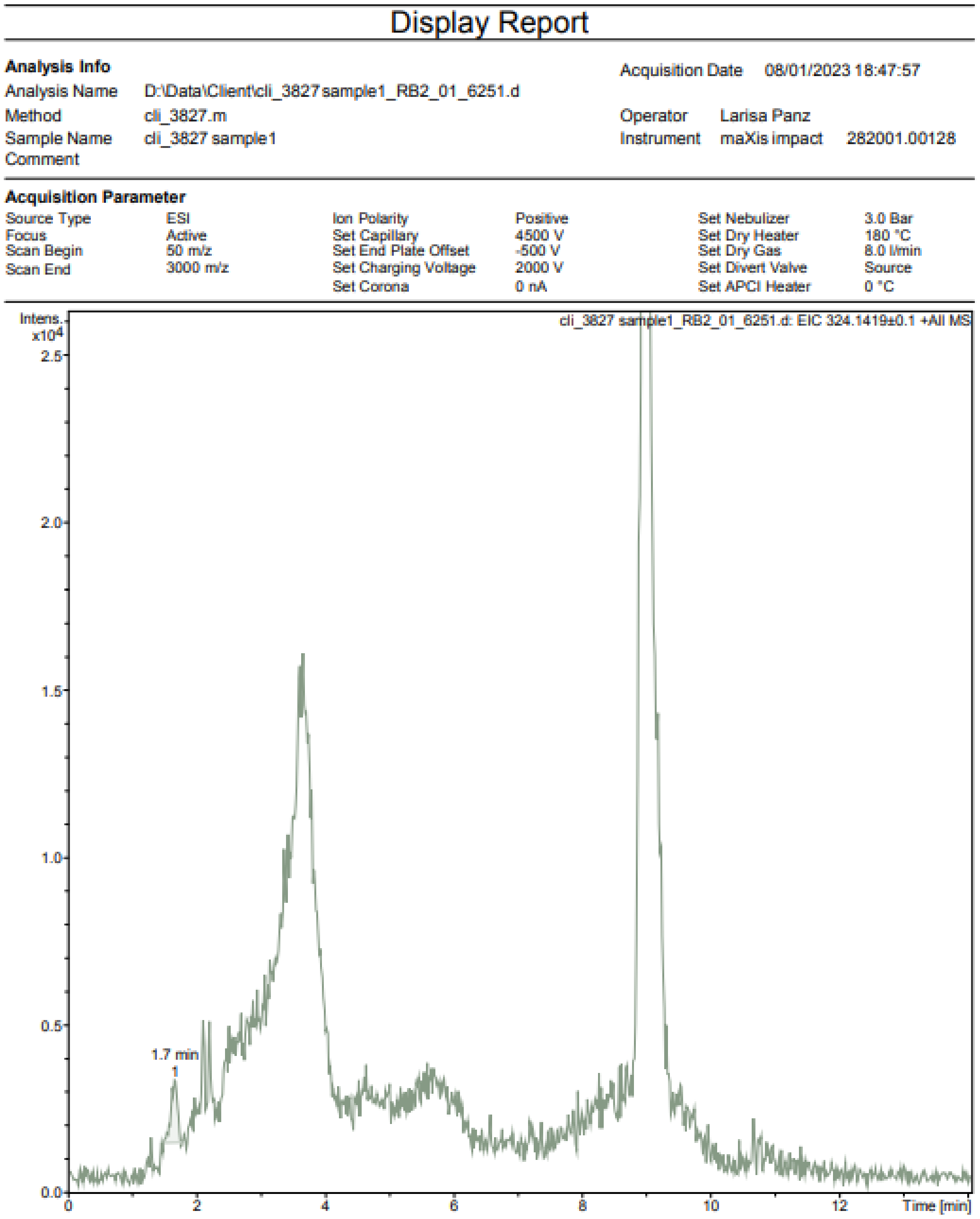

Figure S.27. LC-MS results of the product CMP after reaction of formamide and $CePO_4$ for 48 hours under 170°C and UV irradiation

Display Report

Analysis Info

Analysis Name	D:\Data\Client\cli_3827 sample1_RB2_01_6251.d	Acquisition Date	08/01/2023 18:47:57
Method	cli_3827.m	Operator	Larisa Panz
Sample Name	cli_3827 sample1	Instrument	maXis impact 282001.00128
Comment			

Acquisition Parameter

Source Type	ESI	Ion Polarity	Positive	Set Nebulizer	3.0 Bar
Focus	Active	Set Capillary	4500 V	Set Dry Heater	180 °C
Scan Begin	50 m/z	Set End Plate Offset	-500 V	Set Dry Gas	8.0 l/min
Scan End	3000 m/z	Set Charging Voltage	2000 V	Set Divert Valve	Source
		Set Corona	0 nA	Set APCI Heater	0 °C

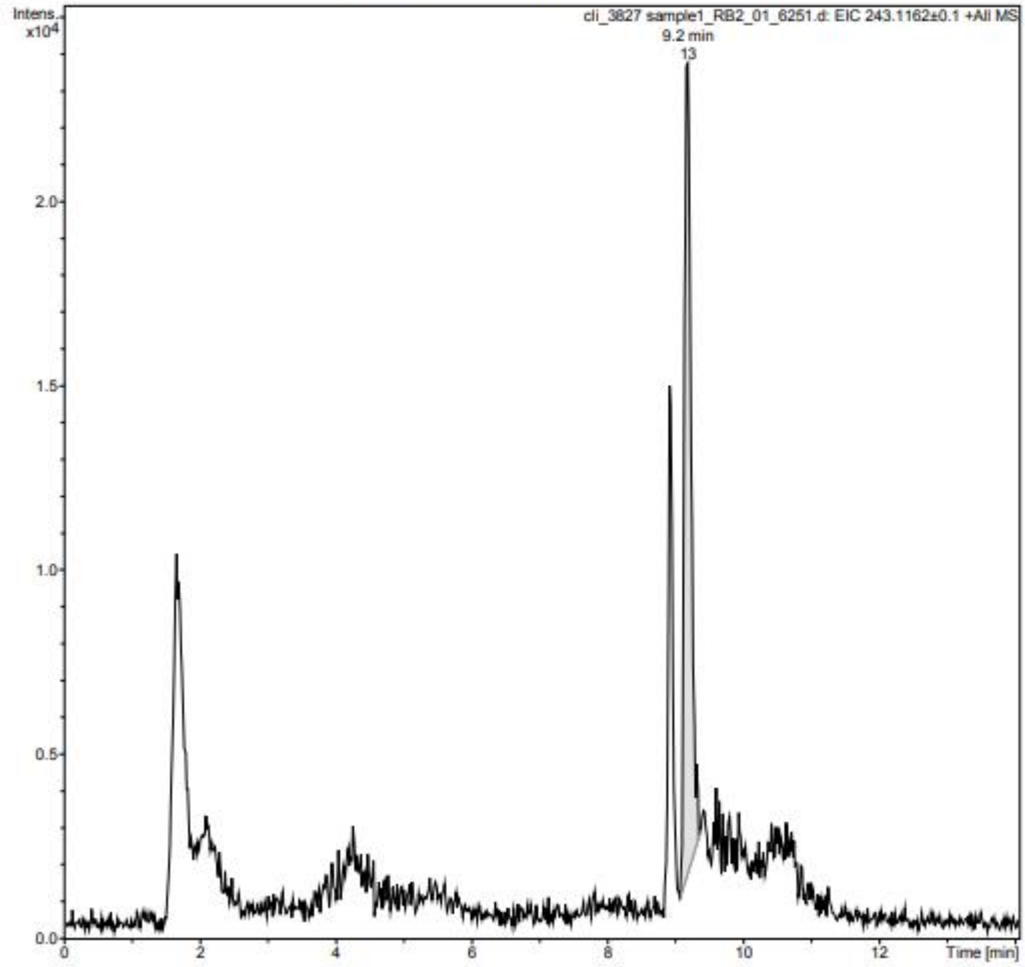

Figure S.28. LC-MS results of the product thymidine after reaction of formamide and $CePO_4$ for 48 hours under 170°C and UV irradiation

Display Report

Analysis Info

Analysis Name	D:\Data\Client\cli_3827sample1_RB2_01_6251.d	Acquisition Date	08/01/2023 18:47:57
Method	cli_3827.m	Operator	Larisa Panz
Sample Name	cli_3827 sample1	Instrument	maXis impact 282001.00128
Comment			

Acquisition Parameter

Source Type	ESI	Ion Polarity	Positive	Set Nebulizer	3.0 Bar
Focus	Active	Set Capillary	4500 V	Set Dry Heater	180 °C
Scan Begin	50 m/z	Set End Plate Offset	-500 V	Set Dry Gas	8.0 l/min
Scan End	3000 m/z	Set Charging Voltage	2000 V	Set Divert Valve	Source
		Set Corona	0 nA	Set APCI Heater	0 °C

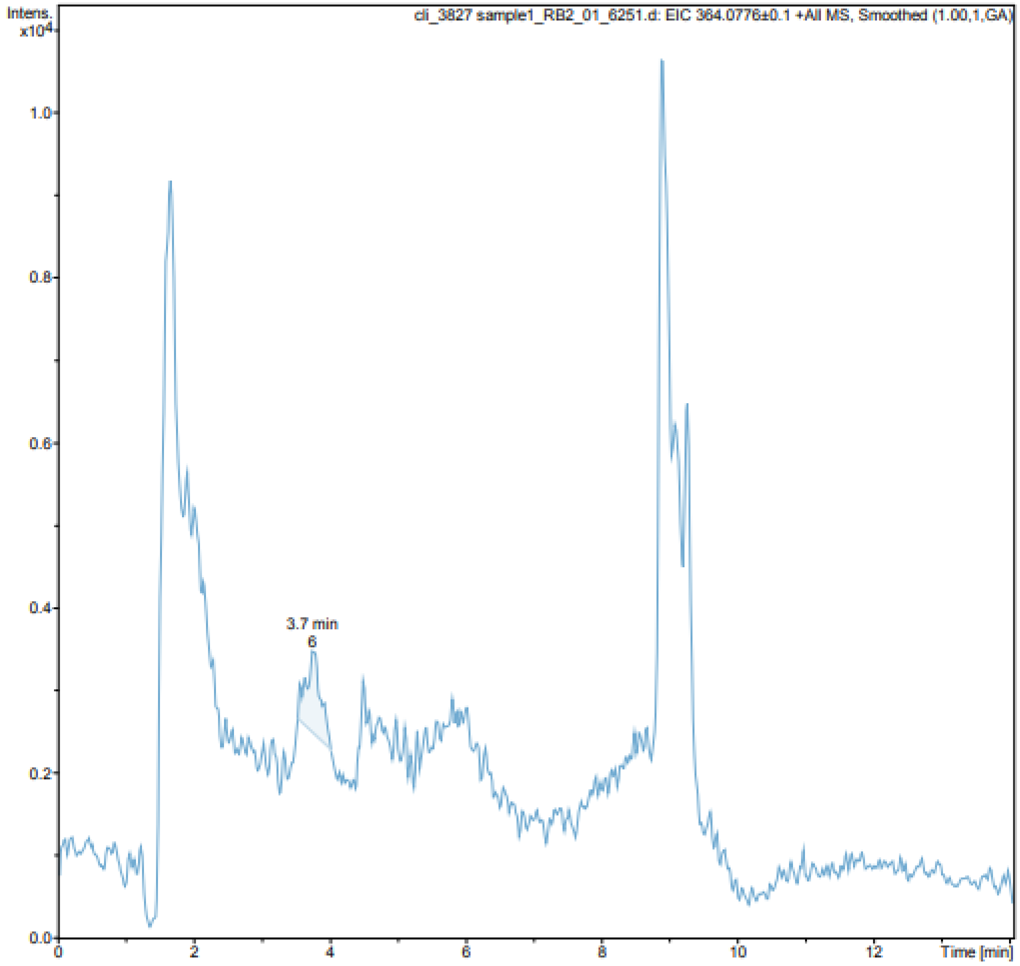

Figure S.29. LC-MS results of the product GMP after reaction of formamide and $CePO_4$ for 48 hours under 170°C and UV irradiation

Display Report

Analysis Info

Analysis Name	D:\Data\Client\cli_3827 sample1_RB2_01_6251.d	Acquisition Date	08/01/2023 18:47:57
Method	cli_3827.m	Operator	Larisa Panz
Sample Name	cli_3827 sample1	Instrument	maXis impact 282001.00128
Comment			

Acquisition Parameter

Source Type	ESI	Ion Polarity	Positive	Set Nebulizer	3.0 Bar
Focus	Active	Set Capillary	4500 V	Set Dry Heater	180 °C
Scan Begin	50 m/z	Set End Plate Offset	-500 V	Set Dry Gas	8.0 l/min
Scan End	3000 m/z	Set Charging Voltage	2000 V	Set Divert Valve	Source
		Set Corona	0 nA	Set APCI Heater	0 °C

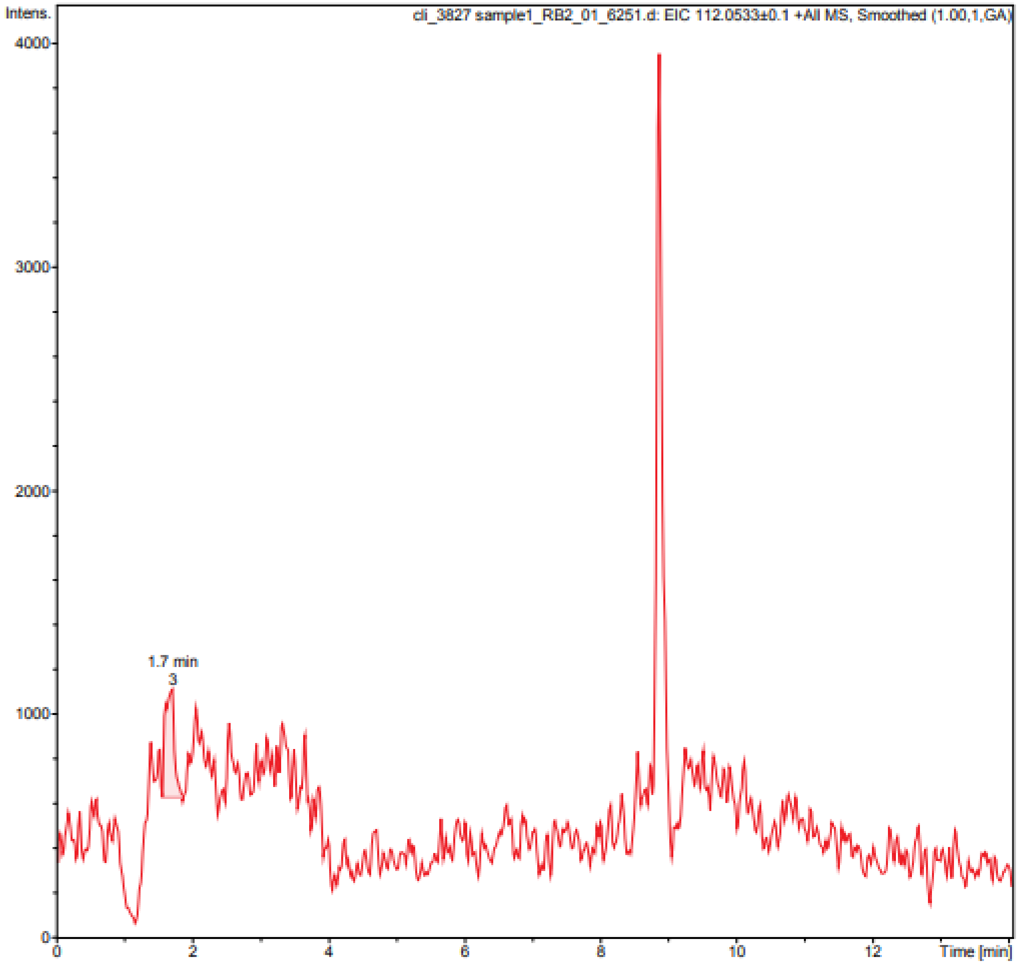

Figure S.30. LC-MS results of the product cytosine after reaction of formamide and $CePO_4$ for 48 hours under 170°C and UV irradiation

Display Report

Analysis Info

Analysis Name	D:\Data\Client\cli_3827 sample1_RB2_01_6251.d	Acquisition Date	08/01/2023 18:47:57
Method	cli_3827.m	Operator	Larisa Panz
Sample Name	cli_3827 sample1	Instrument	maXis impact 282001.00128
Comment			

Acquisition Parameter

Source Type	ESI	Ion Polarity	Positive	Set Nebulizer	3.0 Bar
Focus	Active	Set Capillary	4500 V	Set Dry Heater	180 °C
Scan Begin	50 m/z	Set End Plate Offset	-500 V	Set Dry Gas	8.0 l/min
Scan End	3000 m/z	Set Charging Voltage	2000 V	Set Divert Valve	Source
		Set Corona	0 nA	Set APCI Heater	0 °C

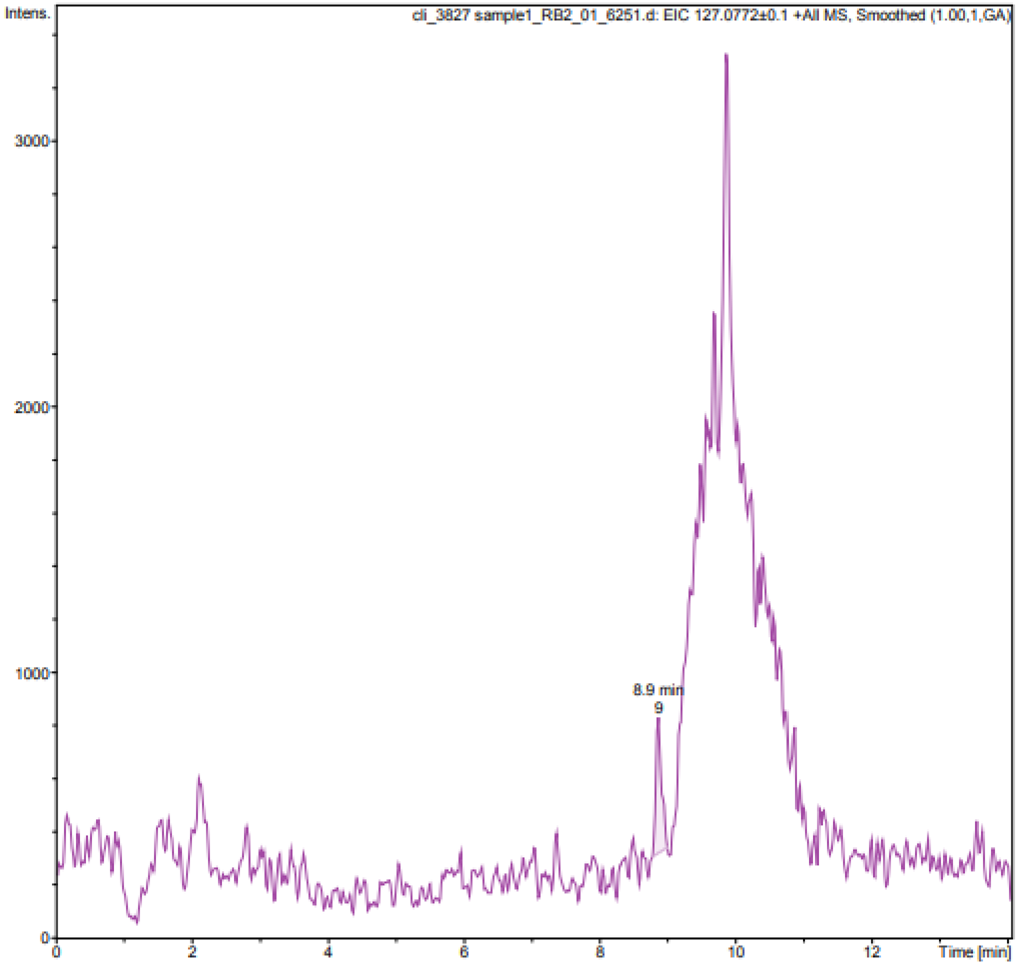

Figure S.33. LC-MS results of the product thymine after reaction of formamide and $CePO_4$ for 48 hours under 170°C and UV irradiation

4. Adsorption experiments

Experimental Details:

Different solutions, each containing one the following compounds (adenine, adenosine, AMP, or cAMP) at a concentration of 3.7mM in formamide were prepared under stirring. 4ml of solution were introduced into a vessel together with 105.2 mg $CePO_4$, yielding a particles' concentration of 2% by weight. The tubes were held in the dark, under stirring and nitrogen flow (0.2 l/m), half of which at a temperature of 300° K and the other half at 333°K. Samples (70µL) were taken at 0, 10, 30, 60, 90, and 120 minutes from the beginning of the process. The samples were centrifuged (10,000 rpm, 10 min.) to separate the liquid from the particulate matter. 25µL of the liquid phase were placed in a clean Eppendorf together with 15µL of formamide and 1.96 ml of HPLC-grade water. The water was added to stabilize the absorption curve of adenine, whose UV-vis absorption spectrum is known to depend on its charge. The UV-vis absorption of said solution was measured using a Shimadzu UV-2600 spectrophotometer to determine the concentration of the species of interest in the solution. All calibration curves were prepared in the same water: formamide ratio (1.96 ml water + 0.04 ml formamide). Table S. 4 describes the composition of each point in the calibration curve.

Table S. 8. The composition of each point in the calibration curve.

Sample name<br>A-Adenine<br>AD-Adenosine<br>AMP- Adenosine mono phosphate<br>cAMP- adenosine cyclic monophosphate	HPLC-grade water	Solution	Formamide
A1/AD1/AMP1/cAMP1	1960µL	5µL	35µL
A2/AD2/AMP2/cAMP2	1960µL	10µL	30µL
A3/AD3/AMP3/cAMP3	1960µL	15µL	25µL
A4/AD4/AMP4/cAMP4	1960µL	20µL	20µL
A5/AD5/AMP5/cAMP5	1960µL	25µL	15µL
A6/AD6/AMP6/cAMP6	1960µL	30µL	1µ0L
A7/AD7/AMP7/cAMP7	1960µL	35µL	5µL
A8/AD8/AMP8/cAMP8	1960µL	40µL	0µL

4A.Calibration Curves

Figures S.32-S.35 show the calibration curves of adenine, adenosine, AMP, and cAMP.

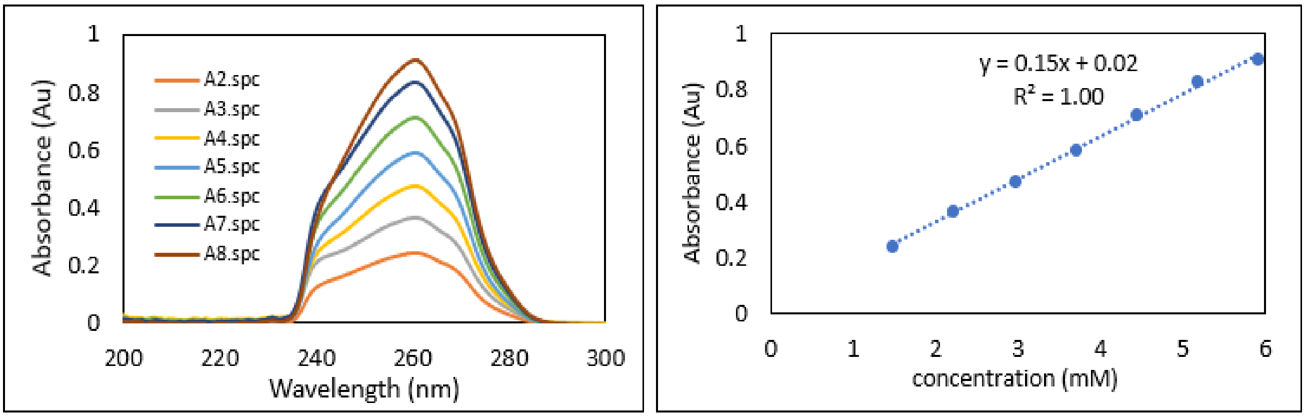

sample	Absorbance (Au)	concentration (mM)
A2	0.24	1.48
A3	0.36	2.22
A4	0.47	2.96
A5	0.59	3.7
A6	0.71	4.44
A7	0.83	5.18
A8	0.91	5.92

Figure S.32. Calibration curve for Adenine (based on absorption at 260.5nm).

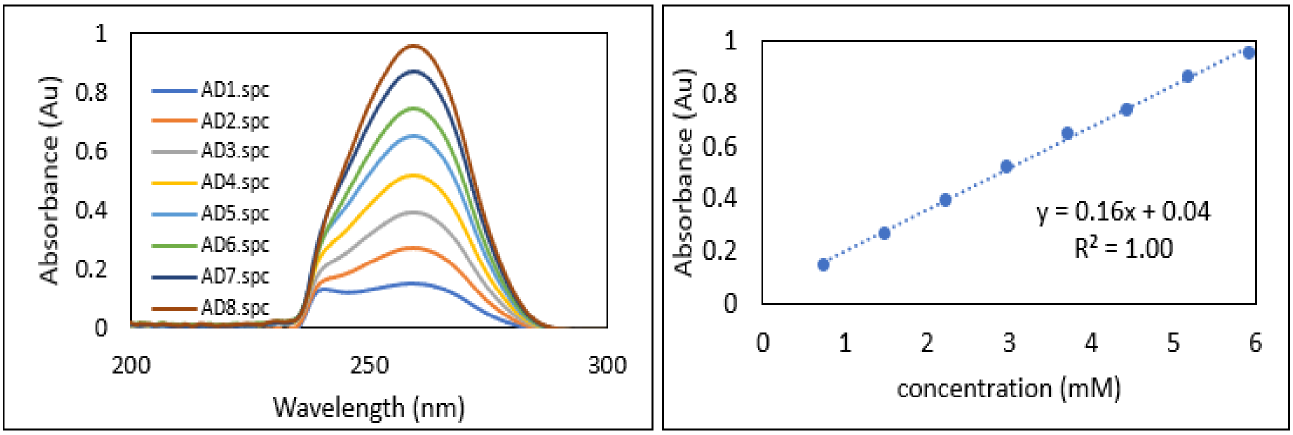

sample	Absorbance (Au)	concentration (mM)
AD1	0.15	0.74
AD2	0.27	1.48
AD3	0.39	2.22
AD4	0.52	2.96
AD5	0.65	3.7
AD6	0.74	4.44
AD7	0.87	5.18
AD8	0.96	5.92

Figure S.34. Calibration curve for Adenosine (based on absorption at 259.5 nm).

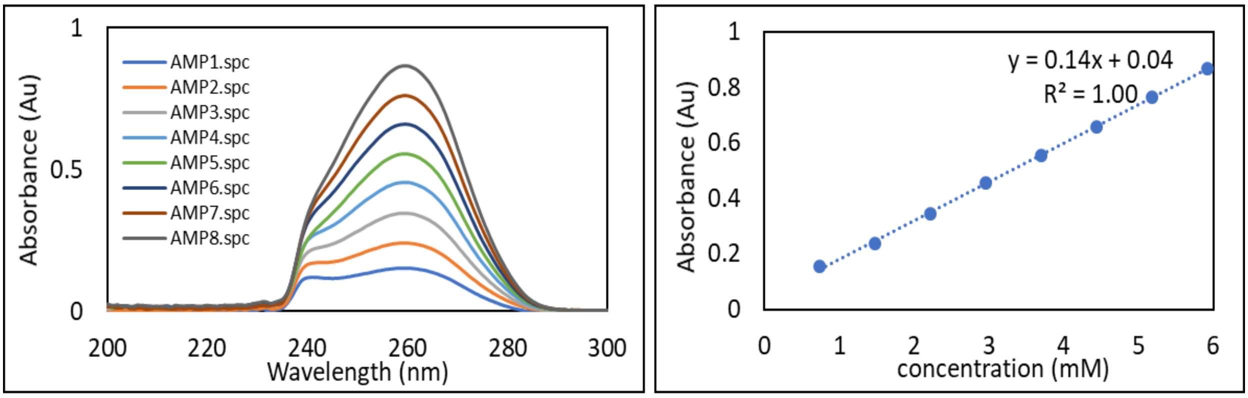

sample	Absorbance (Au)	concentration (mM)
AMP1	0.15	0.74
AMP2	0.24	1.48
AMP3	0.35	2.22
AMP4	0.45	2.96
AMP5	0.56	3.7
AMP6	0.66	4.44
AMP7	0.76	5.18
AMP8	0.87	5.92

Figure S.34. Calibration curve for AMP (based on absorption at 259.5nm).

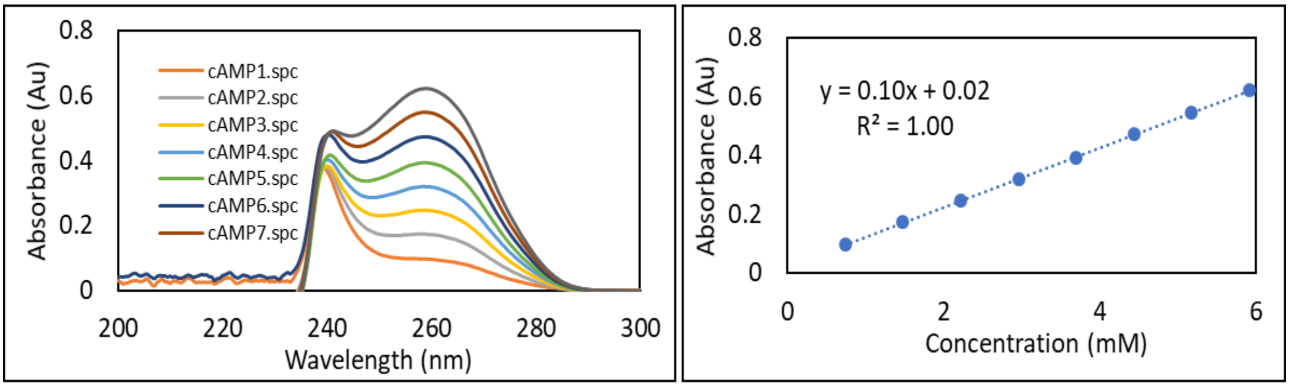

sample	Absorbance (Au)	concentration (mM)
cAMP1	0.10	0.74
cAMP2	0.17	1.48
cAMP3	0.24	2.22
cAMP4	0.32	2.96
cAMP5	0.39	3.7
cAMP6	0.47	4.44
cAMP7	0.55	5.18
cAMP8	0.62	5.92

Figure S.35. Calibration curve for cAMP calibration (based on absorption at 260nm).

4B. Adsorption Kinetics of Adenine, Adenosine, AMP, and cAMP on the Catalyst Surface

Figures S.36 - S.39 show the averaged (n=3) adsorption kinetics of adenine, adenosine, AMP, and cAMP on the catalyst surface at 27°C. In all cases the amount of $CePO_4$ was 105.2 mg.

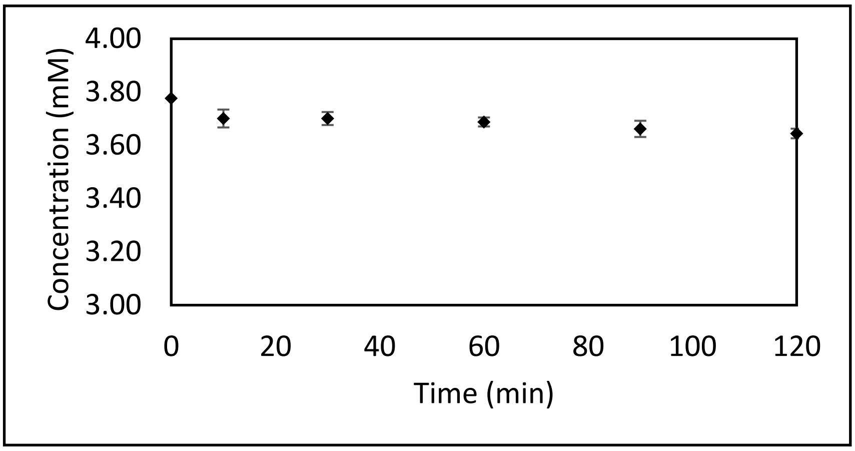

Figure S.36. Adsorption kinetics of adenine on$CePO_4$ at 27°C.

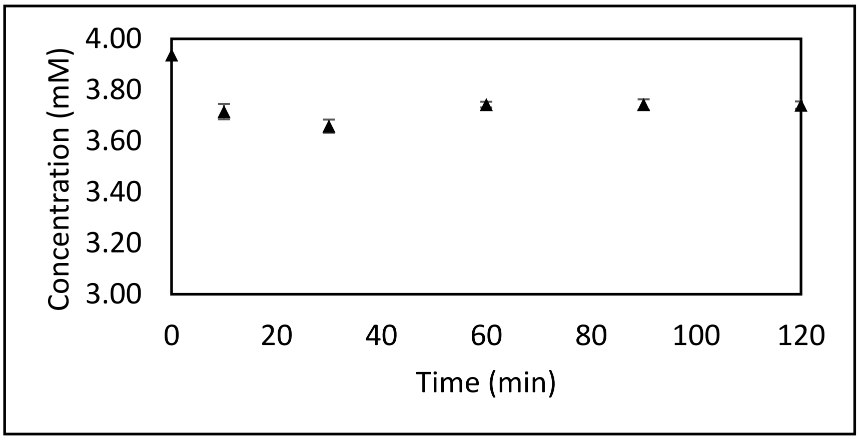

Figure S.37. Adsorption kinetics of adenosine on $CePO_4$ at 27°C.

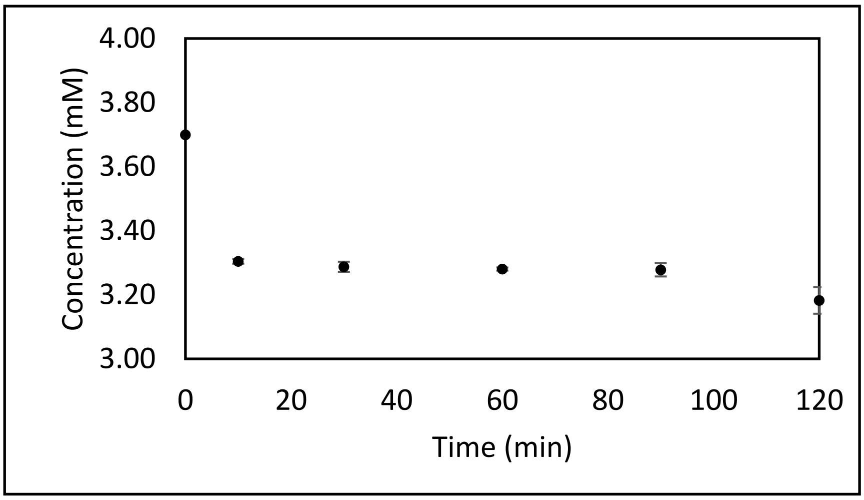

Figure S.38. Adsorption kinetics of AMP on$CePO_4$ at 27°C.

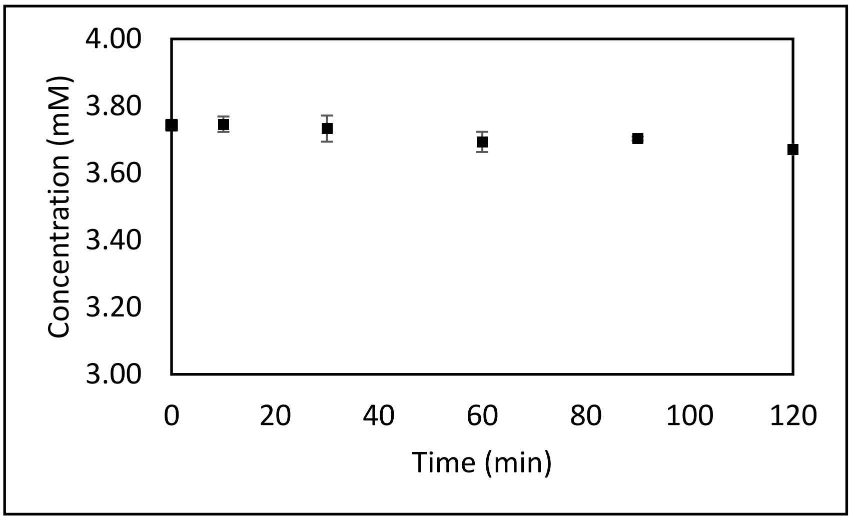

Figure S.39. Adsorption kinetics of cAMP on $CePO_4$ at 27°C.

Table S.5 was prepared based on the results presented in figure S.31-S35. The surface coverage θ was calculated based on the moles of adsorbed species and the area of the catalyst as calculated based on its specific surface area, while considering published data on the average area per molecule.

Table S.5. Calculated surface coverage in the adsorption measurements.

	Area per molecule (nm^2)	Θ (at 300 K)
Adenine	0.805	0.056
Adenosine	1.4	0.139
AMP	1.86	0.48
cAMP	1.55	0.005

5. <u>Change in Turbidity During the Reaction</u>

The change in the absorption spectrum of the reaction solution was monitored along the progression of the reaction in the four types of experiments. Here, 1 ml liquid was centrifuged, with 800 µl of the supernatant diluted in 800 µl formamide prior to measurement. The results are presented in Figures S.36- S.39.

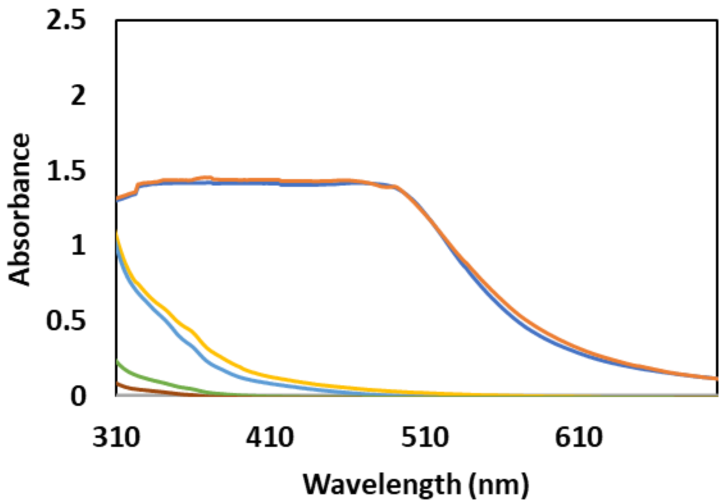

Figure S.40. The change in the absorption along the progression of the reaction in the presence of $CePO_4$ at 170°C and under UV-irradiation, in a duplicate (grey - time 0, green and brown 4h, yellow and cyan 7h, blue and orange 22h).

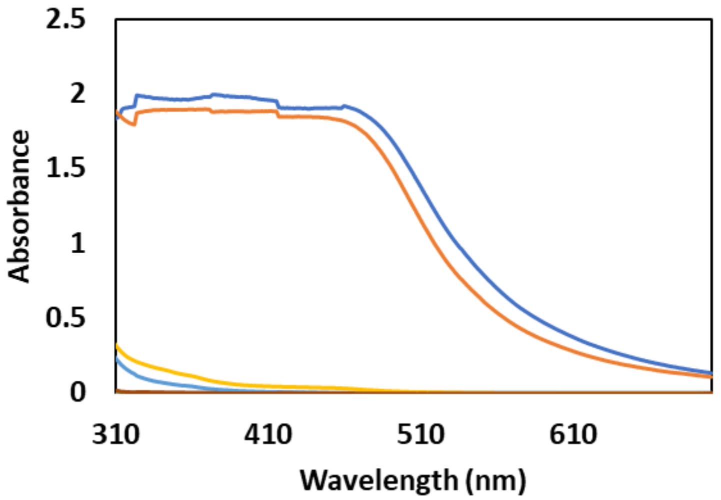

Figure S. 41. The change in the absorption along the progression of the reaction without catalyst at 170°C and under UV-irradiation, in duplicate (grey - time 0, green and brown 4h, yellow and cyan 7h, blue and orange 22h)

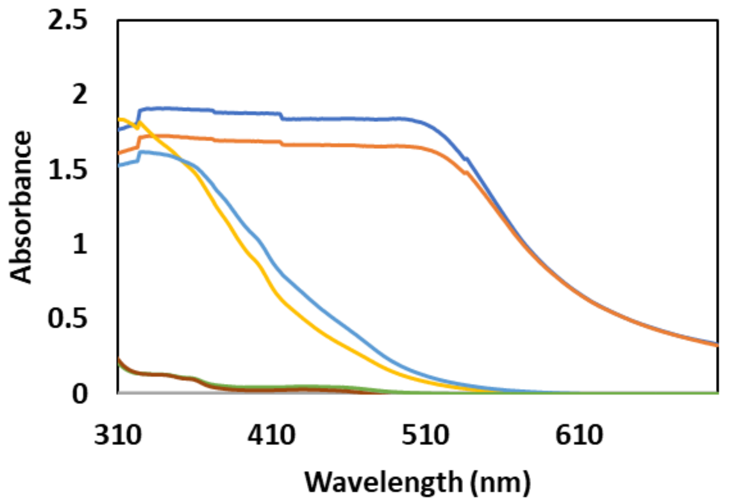

Figure S.42. The change in the absorption along the progression of the reaction formamide without catalyst at 170°C and in the dark, in duplicate (grey - time 0, green and brown 4h, yellow and cyan 7h, blue and orange 22h).

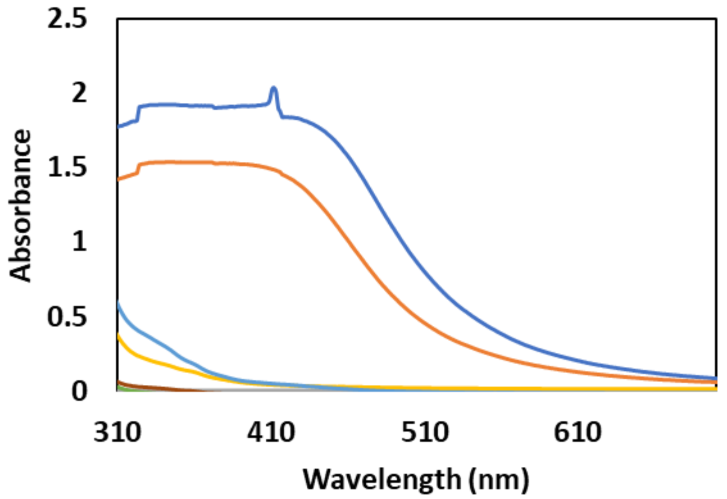

Figure S.43. The change in the absorption along the progression of the reaction formamide in the presence of $CePO_4$ at 170°C and in the dark, in duplicate (grey - time 0, green and brown 4h, yellow and cyan 7h, blue and orange 22h).

<u>**5. Comparison between extract mass LCMS and standards.**</u>

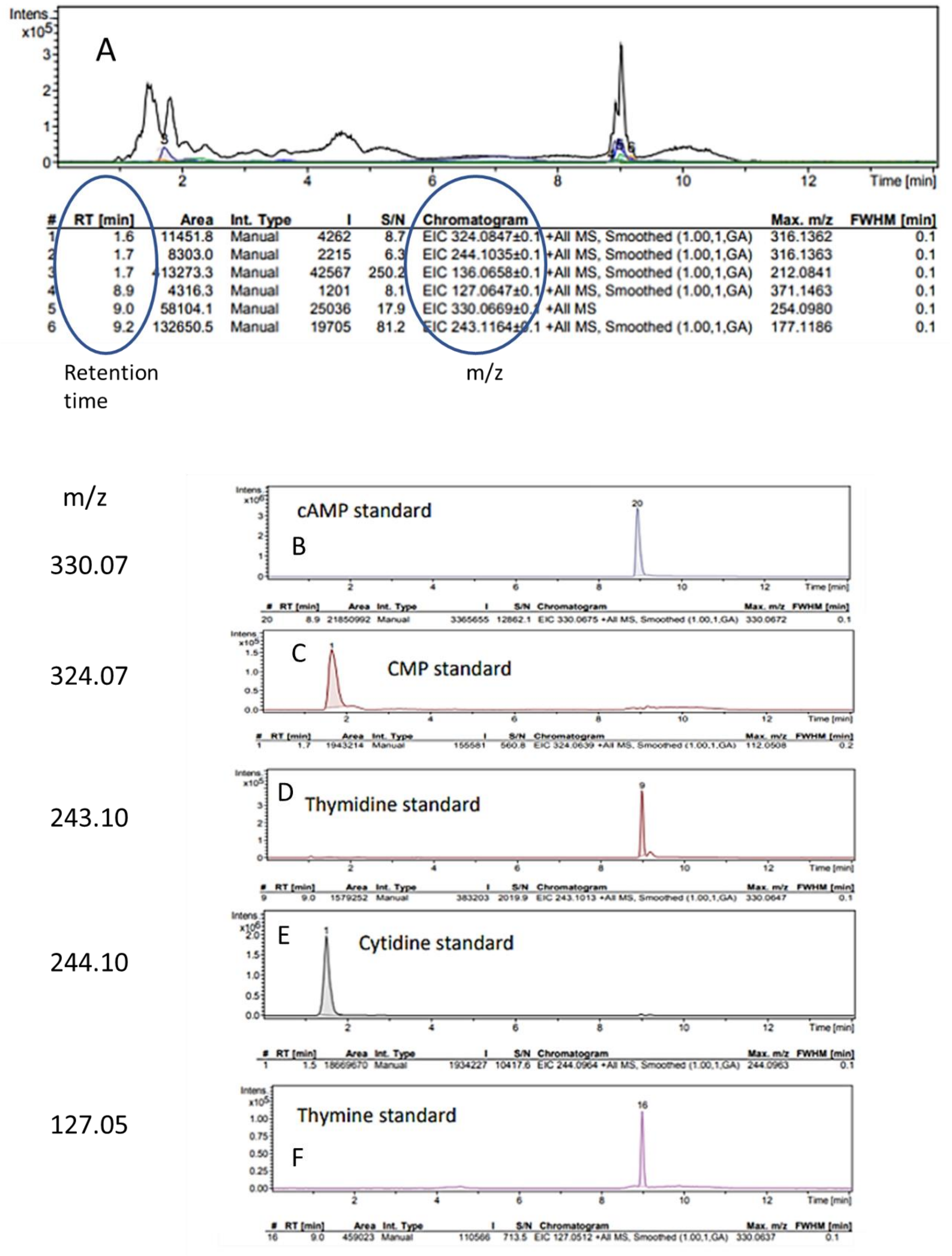

Figure S.44: A comparison between the LC-MS results of one of the samples (CePO4+ light + formamide) [A] and the LC-MS signal of the standards [B-F].

6. XPS Results

The surface of the $CePO_4$ was measured by XPS prior to and after reaction.

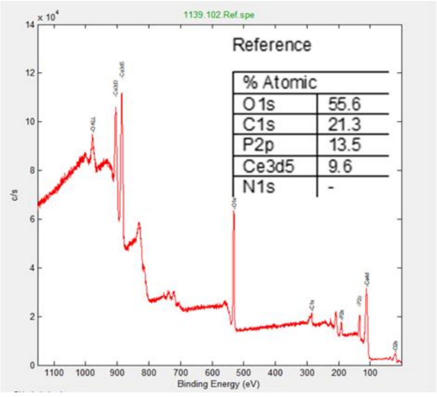

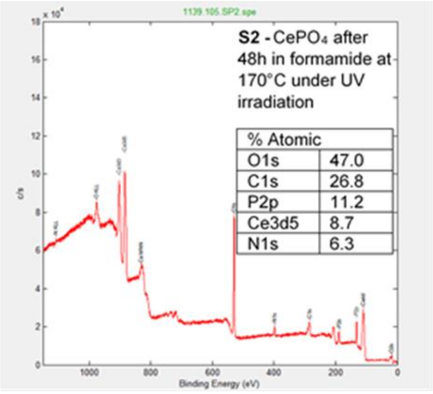

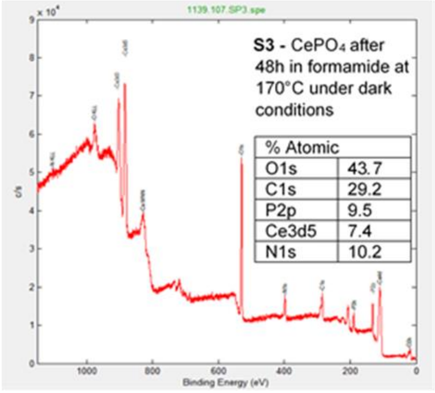

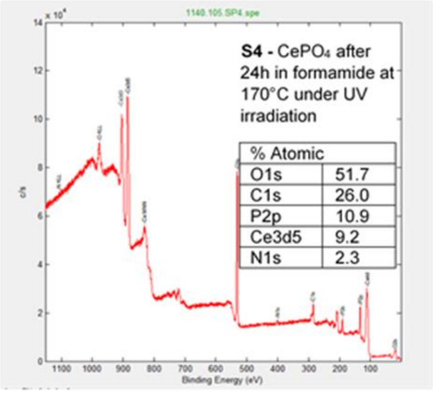

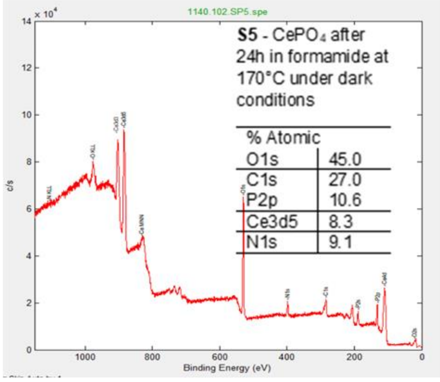

Sample name	P/Ce At% ratio
Reference	1.41
S2	1.29
S3	1.28
S4	1.18
S5	1.28

Figure S.45: XPS results and atomic percentage for: $CePO_4$ prior to reaction (reference), $CePO_4$ following reaction (and washing): after 48 hours under light and in the dark and (S2, S3 respectively) and after 24 hours under light and in the dark (S4, S5 respectively). In the inset table: the ratio between the atomic percentage of cerium to that of phosphorous in the various samples.

Table S.6: Atomic concentrations of O, C, P, Ce, N as obtained by XPS measurements prior to reaction (reference) and following reaction, at various conditions. The ratios between the various elements are also given.

Sample name	% Atomic					% Atomic Ratio				
	O1s	C1s	P2p	Ce3d5	N1s	P/Ce At% ratio	N/Ce At% ratio	N/P At% ratio	O/Ce At% ratio	O/N At% ratio
Prior to reaction	55.6	21.3	13.5	9.6	0	1.41	0	0	5.79	-
$CePO_4$ after 24h in formamide under dark conditions	45.0	27.0	10.6	8.3	9.1	1.28	1.10	0.86	5.42	4.95
$CePO_4$ after 48h in formamide under dark conditions	43.7	29.2	9.5	7.4	10.2	1.28	1.37	1.08	5.90	4.28
$CePO_4$ after 24h in formamide under UV irradiation	51.7	26.0	10.9	9.2	2.3	1.18	0.25	0.21	5.62	22.48
$CePO_4$ after 48h in formamide under UV irradiation	47.0	26.8	11.2	8.7	6.3	1.29	0.72	0.56	5.40	7.46

7. Additional Information

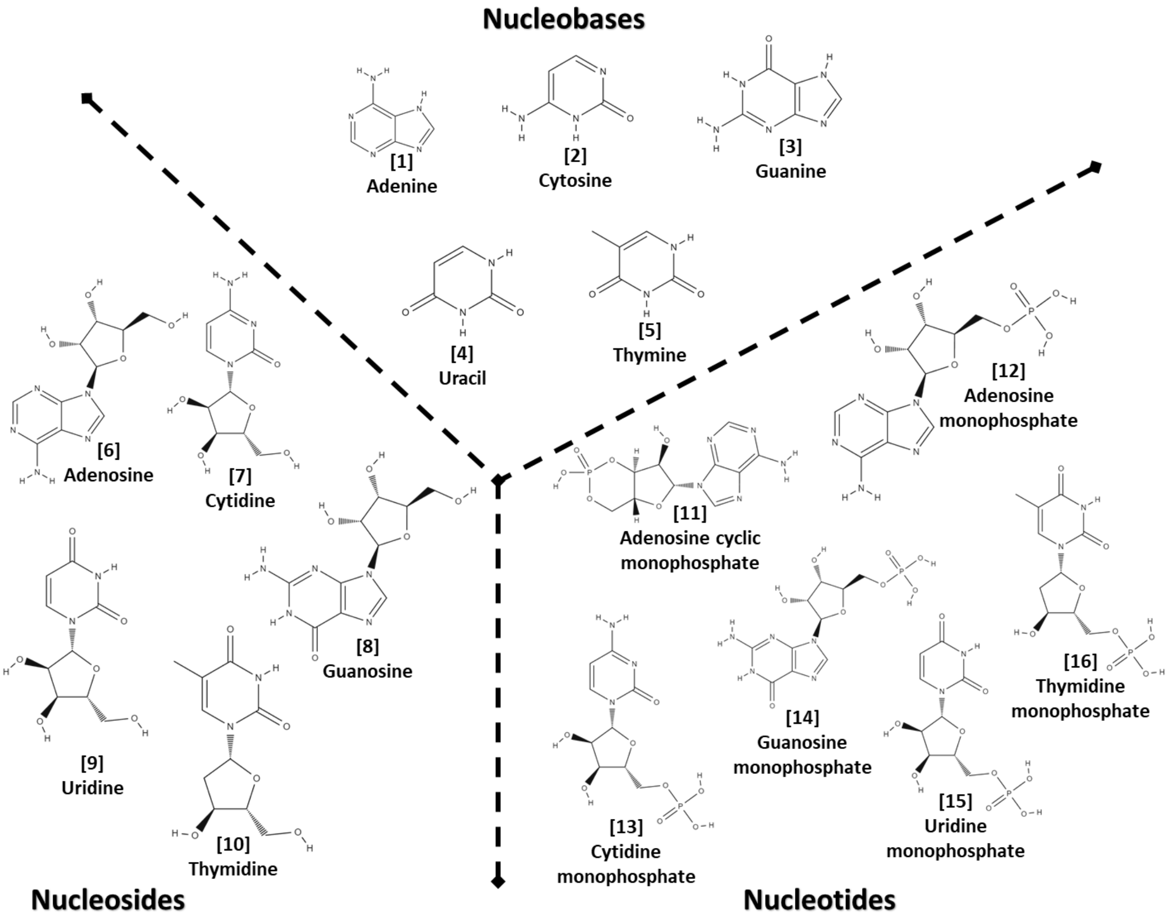

Figure S.46 The various RNA/DNA building blocks discussed in this manuscript

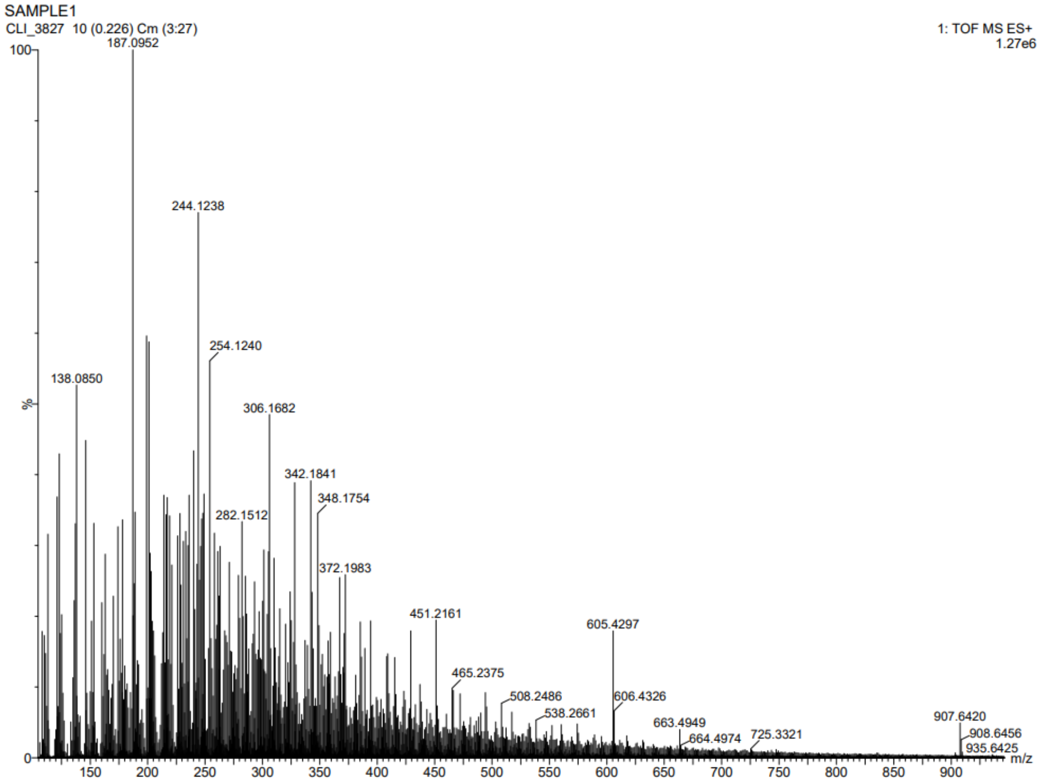

Figure S.47 All detected masses from a high-resolution direct-MS measurement of a sample taken following 48 hours of reaction of formamide at 170°C in the presence of $CePO_4$ and under UV-irradiation.

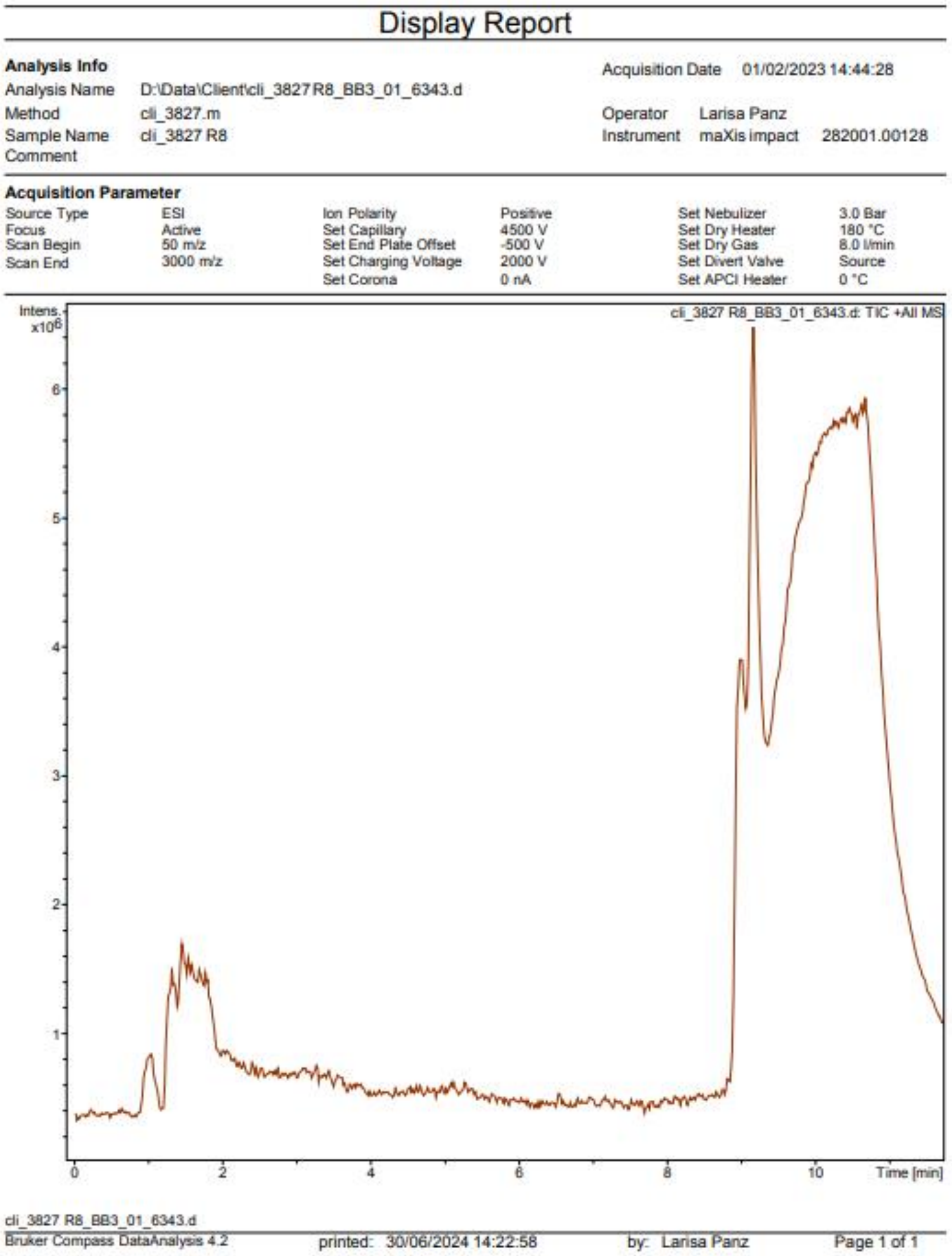

Figure S.48 The Total Ions Current (TIC) signal of the LC-MS following 48 hours of reaction of formamide at 170°C in the presence of $CePO_4$, under dark conditions.

Display Report

Analysis Info

Analysis Name	D:\Data\Client\cli_3827 sample1_RB2_01_6251.d	Acquisition Date	08/01/2023 18:47:57
Method	cli_3827.m	Operator	Larisa Panz
Sample Name	cli_3827 sample1	Instrument	maXis impact 282001.00128
Comment			

Acquisition Parameter

Source Type	ESI	Ion Polarity	Positive	Set Nebulizer	3.0 Bar
Focus	Active	Set Capillary	4500 V	Set Dry Heater	180 °C
Scan Begin	50 m/z	Set End Plate Offset	-500 V	Set Dry Gas	8.0 l/min
Scan End	3000 m/z	Set Charging Voltage	2000 V	Set Divert Valve	Source
		Set Corona	0 nA	Set APCI Heater	0 °C

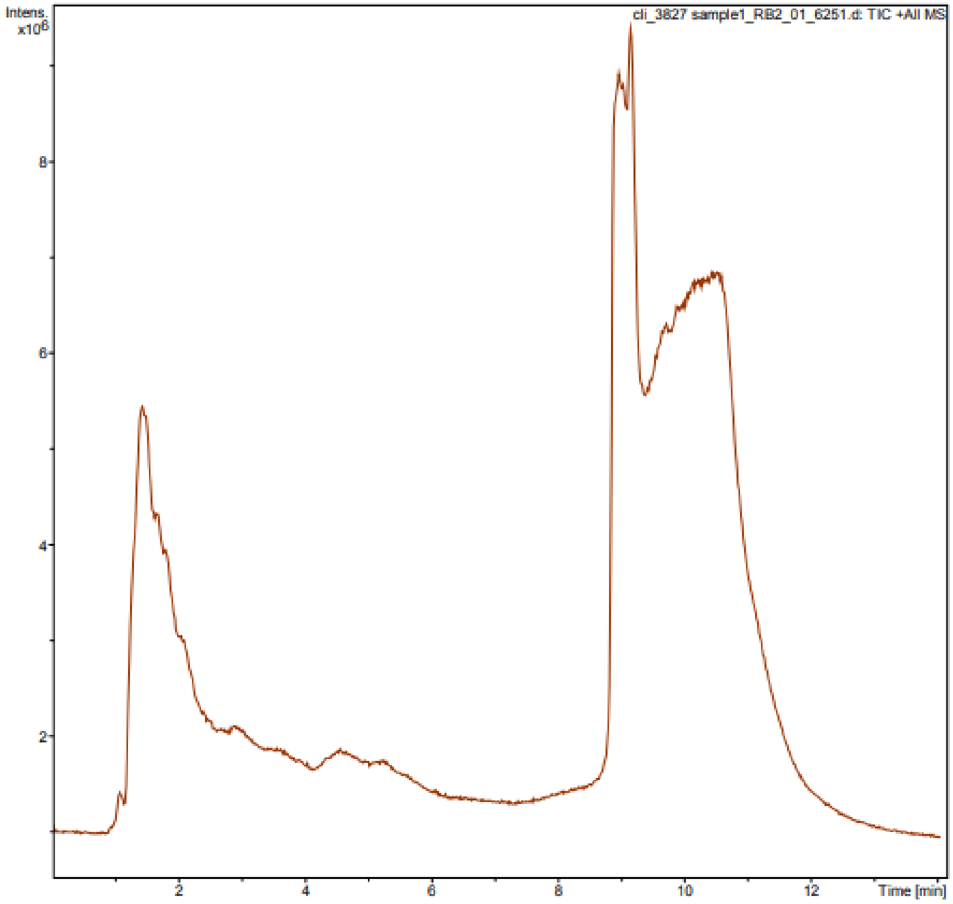

cli_3827 sample1_RB2_01_6251.d

Bruker Compass DataAnalysis 4.2 printed: 30/06/2024 11:28:17 by: Larisa Panz Page 1 of 1

Figure S.49 The Total Ions Current (TIC) signal of the LC-MS following 48 hours of reaction of formamide at 170°C in the presence of $CePO_4$ and under UV-irradiation.
